# Supplementary material for: Thrombosis and thrombocytopenia after vaccination against and infection with SARS-CoV-2 in the United Kingdom
Source: Nat Commun. 2022 Nov 23;13:7167. doi: 10.1038/s41467-022-34668-w (PMC9684520; doi:10.1038/s41467-022-34668-w)
Supplement: Supplementary file 1 — Supplementary Information [file 41467_2022_34668_MOESM1_ESM.pdf]

Supplementary Information

Table of Contents

Supplementary Table 1. Patient characteristics by age group 2

Supplementary Table 2. Results for all study outcomes 11

Supplementary Table 3. Results without requiring year of prior history 20

Supplementary Table 4. Results with general population identified based on a visit/ contact . 30

Supplementary Table 5. Patient profiles: pulmonary embolism 40

Supplementary Table 6. Patient profiles: cerebral venous sinus thrombosis 42

Supplementary Table 7. Patient profiles: thrombocytopenia 44

Supplementary Table 1. Patient characteristics by age group

Characteristics of study participants aged: 20 to 44

The characteristics of the study cohorts used for the primary analyses, all with the requirement to be aged 20 years or older and with a year of prior history observed in the database. Those in the general population were present in the database as of 1 January 2017, while persons infected with SARS-CoV-2 had a confirmatory positive RT-PCR test. *Conditions of interest: autoimmune disease, antiphospholipid syndrome, thrombophilia, asthma, atrial fibrillation, malignant neoplastic disease, diabetes mellitus, obesity, or renal impairment. †Medications of interest included non-steroidal anti-inflammatory drugs, Cox2 inhibitors, systemic corticosteroids, hormonal contraceptives, tamoxifen, and sex hormones and modulators of the genital system

	**ChAdOx1 first dose**	**ChAdOx1 second dose**	**BNT162b2 first dose**	**BNT162b2 second dose**	**SARS-CoV-2 PCR positive test**	**General population**
N	720,734	111,120	338,286	216,637	221,018	4,025,083
Age	36 [30 to 41]	35 [28 to 40]	34 [28 to 40]	34 [28 to 40]	32 [26 to 38]	32 [26 to 38]
Age: 20 to 29	172,501 (23.9%)	31,844 (28.7%)	100,417 (29.7%)	64,580 (29.8%)	89,849 (40.7%)	1,529,282 (38.0%)
Age: 30 to 39	289,137 (40.1%)	48,581 (43.7%)	146,784 (43.4%)	96,100 (44.4%)	89,156 (40.3%)	1,699,641 (42.2%)
Age: 40 to 49	259,096 (35.9%)	30,695 (27.6%)	91,085 (26.9%)	55,957 (25.8%)	42,013 (19.0%)	796,160 (19.8%)
Sex: Male	308,763 (42.8%)	38,706 (34.8%)	111,012 (32.8%)	62,222 (28.7%)	98,848 (44.7%)	2,060,037 (51.2%)
Years of prior observation time	8.3 [3.8 to 17.5]	8.4 [3.7 to 18.7]	7.4 [3.3 to 16.6]	7.2 [3.3 to 16.2]	7.8 [3.3 to 18.6]	7.1 [3.1 to 16.5]
Comorbidities						
Autoimmune disease	14,609 (2.0%)	3,240 (2.9%)	7,313 (2.2%)	4,594 (2.1%)	2,822 (1.3%)	43,353 (1.1%)
Antiphospholipid syndrome	576 (0.1%)	126 (0.1%)	304 (0.1%)	165 (0.1%)	80 (0.0%)	1,350 (0.0%)
Thrombophilia	1,577 (0.2%)	292 (0.3%)	837 (0.2%)	469 (0.2%)	284 (0.1%)	4,336 (0.1%)
Asthma	164,814 (22.9%)	25,955 (23.4%)	73,668 (21.8%)	42,985 (19.8%)	37,710 (17.1%)	616,074 (15.3%)
Atrial fibrillation	1,958 (0.3%)	335 (0.3%)	978 (0.3%)	421 (0.2%)	232 (0.1%)	3,446 (0.1%)

Malignant neoplastic disease	12,582 (1.7%)	3,346 (3.0%)	6,647 (2.0%)	3,830 (1.8%)	1,685 (0.8%)	29,146 (0.7%)
Diabetes mellitus	50,445 (7.0%)	7,820 (7.0%)	24,144 (7.1%)	9,693 (4.5%)	6,908 (3.1%)	79,020 (2.0%)
Obesity	34,333 (4.8%)	5,563 (5.0%)	14,838 (4.4%)	7,846 (3.6%)	6,642 (3.0%)	88,970 (2.2%)
Heart disease	30,746 (4.3%)	5,374 (4.8%)	13,926 (4.1%)	6,944 (3.2%)	4,521 (2.0%)	63,473 (1.6%)
Hypertensive disorder	30,753 (4.3%)	5,346 (4.8%)	14,294 (4.2%)	8,065 (3.7%)	5,718 (2.6%)	80,946 (2.0%)
Renal impairment	9,398 (1.3%)	2,396 (2.2%)	4,734 (1.4%)	2,834 (1.3%)	1,701 (0.8%)	15,134 (0.4%)
COPD	1,512 (0.2%)	455 (0.4%)	670 (0.2%)	323 (0.1%)	106 (0.0%)	2,973 (0.1%)
Dementia	3,274 (0.5%)	326 (0.3%)	963 (0.3%)	441 (0.2%)	375 (0.2%)	4,287 (0.1%)
Medication use (183 days prior to four days prior)						
Non-steroidal anti-inflammatory drugs	58,775 (8.2%)	10,494 (9.4%)	26,234 (7.8%)	15,985 (7.4%)	14,111 (6.4%)	312,181 (7.8%)
Cox2 inhibitors	405 (0.1%)	106 (0.1%)	185 (0.1%)	127 (0.1%)	66 (0.0%)	1,015 (0.0%)
Systemic corticosteroids	30,797 (4.3%)	5,849 (5.3%)	13,910 (4.1%)	8,398 (3.9%)	7,330 (3.3%)	148,169 (3.7%)
Antithrombotic and anticoagulant therapies	2,280 (0.3%)	449 (0.4%)	964 (0.3%)	464 (0.2%)	345 (0.2%)	6,218 (0.2%)
Lipid modifying agents	3,543 (0.5%)	628 (0.6%)	1,673 (0.5%)	707 (0.3%)	547 (0.2%)	8,566 (0.2%)
Antineoplastic and immunomodulating agents	19,149 (2.7%)	4,238 (3.8%)	12,053 (3.6%)	8,457 (3.9%)	6,475 (2.9%)	117,668 (2.9%)
Hormonal contraceptives for systemic use	34,824 (4.8%)	6,587 (5.9%)	20,919 (6.2%)	14,067 (6.5%)	11,162 (5.1%)	190,023 (4.7%)
Tamoxifen	92 (0.0%)	23 (0.0%)	48 (0.0%)	26 (0.0%)	13 (0.0%)	278 (0.0%)
Sex hormones and modulators of the genital system	36,714 (5.1%)	6,966 (6.3%)	21,905 (6.5%)	14,740 (6.8%)	11,618 (5.3%)	197,021 (4.9%)
One or more condition of interest*	112,240 (15.6%)	20,090 (18.1%)	53,013 (15.7%)	26,592 (12.3%)	18,483 (8.4%)	243,685 (6.1%)
One or more medication of interest†	102,002 (14.2%)	18,715 (16.8%)	50,496 (14.9%)	32,138 (14.8%)	27,199 (12.3%)	535,507 (13.3%)

One or more condition/ medication of interest*†	192,651 (26.7%)	34,442 (31.0%)	92,981 (27.5%)	53,321 (24.6%)	42,097 (19.0%)	723,204 (18.0%)

Characteristics of study participants: aged 45 to 64

The characteristics of the study cohorts used for the primary analyses, all with the requirement to be aged 20 years or older and with a year of prior history observed in the database. Those in the general population were present in the database as of 1 January 2017, while persons infected with SARS-CoV-2 had a confirmatory positive RT-PCR test. *Conditions of interest: autoimmune disease, antiphospholipid syndrome, thrombophilia, asthma, atrial fibrillation, malignant neoplastic disease, diabetes mellitus, obesity, or renal impairment. †Medications of interest included non-steroidal anti-inflammatory drugs, Cox2 inhibitors, systemic corticosteroids, hormonal contraceptives, tamoxifen, and sex hormones and modulators of the genital system

	ChAdOx1 first dose	ChAdOx1 second dose	BNT162b2 first dose	BNT162b2 second dose	SARS-CoV-2 PCR positive test	General population
N	1,951,318	238,294	566,080	306,596	143,806	3,204,152
Age	55 [50 to 59]	57 [52 to 61]	56 [51 to 60]	55 [50 to 60]	53 [49 to 58]	54 [49 to 58]
Age: 40 to 49	393,724 (20.2%)	38,163 (16.0%)	108,400 (19.1%)	62,748 (20.5%)	39,956 (27.8%)	879,572 (27.5%)
Age: 50 to 59	1,102,767 (56.5%)	122,937 (51.6%)	295,264 (52.2%)	162,867 (53.1%)	78,377 (54.5%)	1,671,410 (52.2%)
Age: 60 to 69	454,827 (23.3%)	77,194 (32.4%)	162,416 (28.7%)	80,981 (26.4%)	25,473 (17.7%)	653,170 (20.4%)
Sex: Male	1,003,882 (51.4%)	102,149 (42.9%)	229,360 (40.5%)	100,121 (32.7%)	68,965 (48.0%)	1,632,923 (51.0%)
Years of prior observation time	16.1 [7.4 to 25.8]	16.8 [7.6 to 26.8]	16.5 [7.6 to 26.3]	16.6 [7.7 to 26.0]	16.0 [7.4 to 25.4]	15.4 [7.3 to 24.8]
Comorbidities						
Autoimmune disease	45,850 (2.3%)	11,915 (5.0%)	21,359 (3.8%)	12,025 (3.9%)	3,542 (2.5%)	70,764 (2.2%)
Antiphospholipid syndrome	1,343 (0.1%)	376 (0.2%)	709 (0.1%)	363 (0.1%)	130 (0.1%)	1,999 (0.1%)
Thrombophilia	3,269 (0.2%)	737 (0.3%)	1,633 (0.3%)	773 (0.3%)	297 (0.2%)	4,595 (0.1%)
Asthma	258,607 (13.3%)	46,065 (19.3%)	99,253 (17.5%)	51,090 (16.7%)	20,461 (14.2%)	393,138 (12.3%)
Atrial fibrillation	18,033 (0.9%)	5,299 (2.2%)	12,704 (2.2%)	4,768 (1.6%)	1,623 (1.1%)	30,694 (1.0%)
Malignant neoplastic disease	100,340 (5.1%)	25,759 (10.8%)	48,767 (8.6%)	25,813 (8.4%)	7,137 (5.0%)	155,839 (4.9%)

Diabetes mellitus	144,810 (7.4%)	40,842 (17.1%)	92,502 (16.3%)	36,996 (12.1%)	14,576 (10.1%)	241,763 (7.5%)
Obesity	94,273 (4.8%)	19,605 (8.2%)	42,541 (7.5%)	20,458 (6.7%)	9,462 (6.6%)	153,572 (4.8%)
Heart disease	118,274 (6.1%)	29,939 (12.6%)	68,345 (12.1%)	28,300 (9.2%)	10,138 (7.0%)	192,512 (6.0%)
Hypertensive disorder	357,261 (18.3%)	64,637 (27.1%)	143,011 (25.3%)	70,016 (22.8%)	29,205 (20.3%)	566,989 (17.7%)
Renal impairment	52,656 (2.7%)	15,672 (6.6%)	29,178 (5.2%)	14,200 (4.6%)	4,608 (3.2%)	79,184 (2.5%)
COPD	34,389 (1.8%)	15,655 (6.6%)	20,377 (3.6%)	10,974 (3.6%)	2,061 (1.4%)	61,215 (1.9%)
Dementia	4,750 (0.2%)	1,225 (0.5%)	2,054 (0.4%)	858 (0.3%)	368 (0.3%)	5,987 (0.2%)
Medication use (183 days prior to four days prior)						
Non-steroidal anti-inflammatory drugs	181,514 (9.3%)	33,104 (13.9%)	68,965 (12.2%)	35,416 (11.6%)	15,892 (11.1%)	417,991 (13.0%)
Cox2 inhibitors	1,734 (0.1%)	358 (0.2%)	634 (0.1%)	346 (0.1%)	165 (0.1%)	2,802 (0.1%)
Systemic corticosteroids	79,597 (4.1%)	16,380 (6.9%)	30,436 (5.4%)	16,096 (5.2%)	6,451 (4.5%)	180,170 (5.6%)
Antithrombotic and anticoagulant therapies	17,806 (0.9%)	4,998 (2.1%)	11,031 (1.9%)	4,479 (1.5%)	1,524 (1.1%)	40,258 (1.3%)
Lipid modifying agents	50,579 (2.6%)	11,198 (4.7%)	24,777 (4.4%)	10,797 (3.5%)	4,557 (3.2%)	94,537 (3.0%)
Antineoplastic and immunomodulating agents	10,694 (0.5%)	2,977 (1.2%)	5,162 (0.9%)	3,032 (1.0%)	901 (0.6%)	21,562 (0.7%)
Hormonal contraceptives for systemic use	25,489 (1.3%)	3,269 (1.4%)	9,079 (1.6%)	5,769 (1.9%)	2,215 (1.5%)	48,396 (1.5%)
Tamoxifen	858 (0.0%)	199 (0.1%)	452 (0.1%)	233 (0.1%)	60 (0.0%)	1,580 (0.0%)
Sex hormones and modulators of the genital system	57,843 (3.0%)	8,277 (3.5%)	21,661 (3.8%)	13,906 (4.5%)	4,603 (3.2%)	83,088 (2.6%)
One or more condition of interest*	384,475 (19.7%)	93,615 (39.3%)	201,449 (35.6%)	92,974 (30.3%)	34,060 (23.7%)	619,184 (19.3%)

One or more medication of interest[†]	260,141 (13.3%)	46,173 (19.4%)	98,330 (17.4%)	53,133 (17.3%)	22,088 (15.4%)	545,910 (17.0%)
One or more condition/ medication of interest*[†]	569,995 (29.2%)	118,569 (49.8%)	257,996 (45.6%)	125,851 (41.0%)	48,886 (34.0%)	1,007,396 (31.4%)

Characteristics of study participants: age 65 or older

The characteristics of the study cohorts used for the primary analyses, all with the requirement to be aged 20 years or older and with a year of prior history observed in the database. Those in the general population were present in the database as of 1 January 2017, while persons infected with SARS-CoV-2 had a confirmatory positive RT-PCR test. *Conditions of interest: autoimmune disease, antiphospholipid syndrome, thrombophilia, asthma, atrial fibrillation, malignant neoplastic disease, diabetes mellitus, obesity, or renal impairment. †Medications of interest included non-steroidal anti-inflammatory drugs, Cox2 inhibitors, systemic corticosteroids, hormonal contraceptives, tamoxifen, and sex hormones and modulators of the genital system

	**ChAdOx1 first dose**	**ChAdOx1 second dose**	**BNT162b2 first dose**	**BNT162b2 second dose**	**SARS-CoV-2 PCR positive test**	**General population**
N	1,096,465	742,246	928,475	778,761	36,867	2,185,168
Age	72 [68 to 77]	74 [70 to 78]	77 [72 to 83]	78 [73 to 84]	72 [67 to 79]	74 [69 to 81]
Age: 60 to 69	358,850 (32.7%)	148,747 (20.0%)	165,623 (17.8%)	100,706 (12.9%)	14,070 (38.2%)	601,933 (27.5%)
Age: 70 to 79	558,629 (50.9%)	445,900 (60.1%)	375,954 (40.5%)	333,504 (42.8%)	14,312 (38.8%)	960,021 (43.9%)
Age: 80 or older	178,986 (16.3%)	147,599 (19.9%)	386,898 (41.7%)	344,551 (44.2%)	8,485 (23.0%)	623,214 (28.5%)
Sex: Male	510,982 (46.6%)	340,397 (45.9%)	424,994 (45.8%)	353,322 (45.4%)	17,003 (46.1%)	1,004,458 (46.0%)
Years of prior observation time	23.5 [10.0 to 34.5]	24.1 [10.4 to 35.1]	24.9 [11.4 to 35.6]	25.2 [11.6 to 36.1]	21.2 [7.5 to 33.0]	22.7 [11.0 to 33.3]
Comorbidities	NA	NA	NA	NA	NA	NA
Autoimmune disease	44,457 (4.1%)	32,390 (4.4%)	39,600 (4.3%)	34,021 (4.4%)	1,618 (4.4%)	81,940 (3.7%)
Antiphospholipid syndrome	500 (0.0%)	353 (0.0%)	443 (0.0%)	362 (0.0%)	14 (0.0%)	785 (0.0%)
Thrombophilia	1,286 (0.1%)	880 (0.1%)	1,046 (0.1%)	864 (0.1%)	36 (0.1%)	1,883 (0.1%)
Asthma	135,943 (12.4%)	94,426 (12.7%)	115,241 (12.4%)	96,991 (12.5%)	5,281 (14.3%)	248,992 (11.4%)
Atrial fibrillation	90,924 (8.3%)	70,429 (9.5%)	107,757 (11.6%)	94,794 (12.2%)	3,700 (10.0%)	197,215 (9.0%)

Malignant neoplastic disease	206,533 (18.8%)	156,554 (21.1%)	222,906 (24.0%)	194,735 (25.0%)	6,749 (18.3%)	421,038 (19.3%)
Diabetes mellitus	187,504 (17.1%)	132,517 (17.9%)	170,270 (18.3%)	143,448 (18.4%)	8,234 (22.3%)	363,293 (16.6%)
Obesity	61,884 (5.6%)	41,958 (5.7%)	47,268 (5.1%)	38,684 (5.0%)	2,679 (7.3%)	104,761 (4.8%)
Heart disease	263,163 (24.0%)	196,810 (26.5%)	282,054 (30.4%)	244,146 (31.4%)	10,611 (28.8%)	568,105 (26.0%)
Hypertensive disorder	526,949 (48.1%)	379,691 (51.2%)	507,925 (54.7%)	435,278 (55.9%)	19,210 (52.1%)	1,113,255 (50.9%)
Renal impairment	180,000 (16.4%)	139,302 (18.8%)	210,451 (22.7%)	185,328 (23.8%)	7,587 (20.6%)	417,433 (19.1%)
COPD	83,381 (7.6%)	61,943 (8.3%)	77,084 (8.3%)	65,976 (8.5%)	3,010 (8.2%)	169,253 (7.7%)
Dementia	37,453 (3.4%)	29,755 (4.0%)	33,803 (3.6%)	29,668 (3.8%)	3,309 (9.0%)	85,660 (3.9%)
Medication use (183 days prior to four days prior)	NA	NA	NA	NA	NA	NA
Non-steroidal anti-inflammatory drugs	173,279 (15.8%)	123,500 (16.6%)	161,995 (17.4%)	136,857 (17.6%)	7,846 (21.3%)	482,673 (22.1%)
Cox2 inhibitors	999 (0.1%)	680 (0.1%)	838 (0.1%)	713 (0.1%)	42 (0.1%)	2,085 (0.1%)
Systemic corticosteroids	76,515 (7.0%)	55,331 (7.5%)	69,406 (7.5%)	59,289 (7.6%)	3,139 (8.5%)	209,028 (9.6%)
Antithrombotic and anticoagulant therapies	50,500 (4.6%)	37,742 (5.1%)	54,856 (5.9%)	47,596 (6.1%)	1,958 (5.3%)	143,580 (6.6%)
Lipid modifying agents	83,805 (7.6%)	60,134 (8.1%)	78,656 (8.5%)	67,434 (8.7%)	3,163 (8.6%)	187,590 (8.6%)
Antineoplastic and immunomodulating agents	9,744 (0.9%)	7,240 (1.0%)	9,788 (1.1%)	8,550 (1.1%)	321 (0.9%)	22,192 (1.0%)
Hormonal contraceptives for systemic use	1,096 (0.1%)	608 (0.1%)	758 (0.1%)	585 (0.1%)	32 (0.1%)	2,324 (0.1%)

Tamoxifen	331 (0.0%)	224 (0.0%)	317 (0.0%)	274 (0.0%)	12 (0.0%)	892 (0.0%)
Sex hormones and modulators of the genital system	15,233 (1.4%)	10,501 (1.4%)	12,359 (1.3%)	10,450 (1.3%)	442 (1.2%)	24,619 (1.1%)
One or more condition of interest*	533,098 (48.6%)	390,092 (52.6%)	529,613 (57.0%)	455,052 (58.4%)	20,390 (55.3%)	1,093,235 (50.0%)
One or more medication of interest†	214,600 (19.6%)	153,120 (20.6%)	197,803 (21.3%)	167,518 (21.5%)	9,313 (25.3%)	569,004 (26.0%)
One or more condition/ medication of interest*†	623,301 (56.8%)	450,385 (60.7%)	600,672 (64.7%)	513,412 (65.9%)	23,805 (64.6%)	1,331,667 (60.9%)

Supplementary Table 2. Results for all study outcomes

For each event of interest the number of persons contributing to the analysis from the target population, their person-years contributed, and the number of events observed for them are given. Their expected events are estimated using indirect standardisation to the general population, with expected events giving the number of events we would have expected to have seen if their outcome experience was the same as that of the general population. Standardised incidence ratios (SIRs) with 95% confidence intervals (CIs) were estimated. Events with less than 5 occurrences have been omitted for privacy reasons.

Event	Cohort	N	Person-years	Observed events	Expected events	SIR (95% CI)
Cerebral venous sinus thrombosis	ChAdOx1 first dose	3,764,507	277,866	16	3.9	4.14 (2.54 to 6.76)
Cerebral venous sinus thrombosis	SARS-CoV-2 PCR positive test	401,568	95,737	5	1.3	3.74 (1.56 to 8.98)
Deep vein thrombosis - broad	ChAdOx1 first dose	3,756,629	277,248	555	505.4	1.10 (1.01 to 1.19)
Deep vein thrombosis - broad	ChAdOx1 second dose	1,087,452	57,778	139	156.5	0.89 (0.75 to 1.05)
Deep vein thrombosis - broad	BNT162b2 first dose	1,826,442	139,055	353	336.6	1.05 (0.94 to 1.16)
Deep vein thrombosis - broad	BNT162b2 second dose	1,297,754	86,102	220	235.1	0.94 (0.82 to 1.07)
Deep vein thrombosis - broad	SARS-CoV-2 PCR positive test	401,035	95,569	299	104.2	2.87 (2.56 to 3.21)
Deep vein thrombosis - narrow	ChAdOx1 first dose	3,757,806	277,341	456	448.5	1.02 (0.93 to 1.11)
Deep vein thrombosis - narrow	ChAdOx1 second dose	1,087,890	57,802	121	141.1	0.86 (0.72 to 1.02)

Deep vein thrombosis - narrow	BNT162b2 first dose	1,827,104	139,108	303	303.9	1.00 (0.89 to 1.12)
Deep vein thrombosis - narrow	BNT162b2 second dose	1,298,221	86,134	182	213.6	0.85 (0.74 to 0.99)
Deep vein thrombosis - narrow	SARS-CoV-2 PCR positive test	401,111	95,592	265	91.2	2.91 (2.58 to 3.28)
Deep vein thrombosis broad with thrombocytopenia 10 days pre to 10 days post	ChAdOx1 first dose	3,764,541	277,868	16	7.9	2.03 (1.24 to 3.31)
Deep vein thrombosis broad with thrombocytopenia 10 days pre to 10 days post	BNT162b2 second dose	1,301,213	86,338	8	3.9	2.05 (1.03 to 4.11)
Deep vein thrombosis broad with thrombocytopenia 42 days pre to 14 days post	ChAdOx1 first dose	3,764,502	277,865	21	12.3	1.71 (1.12 to 2.63)
Deep vein thrombosis broad with thrombocytopenia 42 days pre to 14 days post	BNT162b2 first dose	1,831,401	139,447	7	8.9	0.79 (0.38 to 1.65)
Deep vein thrombosis broad with thrombocytopenia 42 days pre to 14 days post	BNT162b2 second dose	1,301,178	86,336	12	6.4	1.89 (1.07 to 3.33)
Deep vein thrombosis narrow with thrombocytopenia 10 days pre to 10 days post	ChAdOx1 first dose	3,764,546	277,869	11	7.4	1.49 (0.83 to 2.69)
Deep vein thrombosis narrow with thrombocytopenia 10 days pre to 10 days post	BNT162b2 second dose	1,301,218	86,339	8	3.6	2.24 (1.12 to 4.47)
Deep vein thrombosis narrow with thrombocytopenia 42 days pre to 14 days post	ChAdOx1 first dose	3,764,509	277,866	16	11.5	1.40 (0.86 to 2.28)
Deep vein thrombosis narrow with thrombocytopenia 42 days pre to 14 days post	BNT162b2 first dose	1,831,409	139,447	7	8.3	0.85 (0.40 to 1.77)
Deep vein thrombosis narrow with thrombocytopenia 42 days pre to 14 days post	BNT162b2 second dose	1,301,185	86,336	11	5.9	1.85 (1.03 to 3.35)
Hemorrhagic stroke	ChAdOx1 first dose	3,763,357	277,777	74	90.3	0.82 (0.65 to 1.03)
Hemorrhagic stroke	ChAdOx1 second dose	1,090,433	57,943	20	30.2	0.66 (0.43 to 1.03)

Hemorrhagic stroke	BNT162b2 first dose	1,830, 843	139,403	45	66.8	0.67 (0.50 to 0.90)
Hemorrhagic stroke	BNT162b2 second dose	1,300, 839	86,313	29	48.4	0.60 (0.42 to 0.86)
Hemorrhagic stroke	SARS-CoV-2 PCR positive test	401,47 1	95,713	20	17.9	1.12 (0.72 to 1.73)
Immune thrombocytopenia	ChAdOx1 first dose	3,764, 198	277,841	45	25.2	1.79 (1.33 to 2.39)
Immune thrombocytopenia	ChAdOx1 second dose	1,090, 713	57,959	8	7.7	1.04 (0.52 to 2.07)
Immune thrombocytopenia	BNT162b2 first dose	1,831, 194	139,430	21	16.4	1.28 (0.83 to 1.96)
Immune thrombocytopenia	BNT162b2 second dose	1,301, 033	86,326	9	11.4	0.79 (0.41 to 1.52)
Immune thrombocytopenia	SARS-CoV-2 PCR positive test	401,53 9	95,729	10	5.9	1.70 (0.92 to 3.17)
Intestinal infarction	ChAdOx1 first dose	3,764, 223	277,844	25	24.2	1.03 (0.70 to 1.53)
Intestinal infarction	ChAdOx1 second dose	1,090, 726	57,959	5	8.3	0.60 (0.25 to 1.45)
Intestinal infarction	BNT162b2 first dose	1,831, 256	139,435	17	17	1.00 (0.62 to 1.61)
Intestinal infarction	BNT162b2 second dose	1,301, 091	86,331	9	12.1	0.75 (0.39 to 1.43)
Intestinal infarction	SARS-CoV-2 PCR positive test	401,54 7	95,732	7	4.2	1.65 (0.79 to 3.47)
Ischemic stroke	ChAdOx1 first dose	3,762, 624	277,719	128	155.8	0.82 (0.69 to 0.98)
Ischemic stroke	ChAdOx1 second dose	1,089, 880	57,912	47	63.3	0.74 (0.56 to 0.99)

Ischemic stroke	BNT162b2 first dose	1,830,001	139,335	146	132.9	1.10 (0.93 to 1.29)
Ischemic stroke	BNT162b2 second dose	1,300,162	86,264	68	99.9	0.68 (0.54 to 0.86)
Ischemic stroke	SARS-CoV-2 PCR positive test	401,463	95,710	28	23.2	1.21 (0.83 to 1.75)
Mesenteric vein thrombosis	ChAdOx1 first dose	3,764,576	277,871	5	3.4	1.46 (0.61 to 3.50)
Myocardial infarction	ChAdOx1 first dose	3,755,717	277,176	606	708.6	0.86 (0.79 to 0.93)
Myocardial infarction	ChAdOx1 second dose	1,086,394	57,724	166	238.1	0.70 (0.60 to 0.81)
Myocardial infarction	BNT162b2 first dose	1,824,272	138,887	442	500.6	0.88 (0.80 to 0.97)
Myocardial infarction	BNT162b2 second dose	1,296,561	86,025	283	355.8	0.80 (0.71 to 0.89)
Myocardial infarction	SARS-CoV-2 PCR positive test	400,986	95,580	167	114.4	1.46 (1.25 to 1.70)
Myocardial infarction or ischemic stroke	ChAdOx1 first dose	3,752,849	276,954	712	839.2	0.85 (0.79 to 0.91)
Myocardial infarction or ischemic stroke	ChAdOx1 second dose	1,084,976	57,646	209	292.9	0.71 (0.62 to 0.82)
Myocardial infarction or ischemic stroke	BNT162b2 first dose	1,822,051	138,713	568	616	0.92 (0.85 to 1.00)
Myocardial infarction or ischemic stroke	BNT162b2 second dose	1,295,041	85,920	344	443.6	0.78 (0.70 to 0.86)
Myocardial infarction or ischemic stroke	SARS-CoV-2 PCR positive test	400,818	95,538	186	133.5	1.39 (1.21 to 1.61)
Myocardial infarction or ischemic stroke with thrombocytopenia 10 days pre to 10 days post	ChAdOx1 first dose	3,764,535	277,868	7	11.5	0.61 (0.29 to 1.27)

Myocardial infarction or ischemic stroke with thrombocytopenia 10 days pre to 10 days post	BNT162b2 first dose	1,831,410	139,447	6	9.8	0.61 (0.27 to 1.36)
Myocardial infarction or ischemic stroke with thrombocytopenia 42 days pre to 14 days post	ChAdOx1 first dose	3,764,471	277,863	12	18.6	0.65 (0.37 to 1.14)
Myocardial infarction or ischemic stroke with thrombocytopenia 42 days pre to 14 days post	BNT162b2 first dose	1,831,361	139,443	11	15.9	0.69 (0.38 to 1.25)
Myocardial infarction or ischemic stroke with thrombocytopenia 42 days pre to 14 days post	BNT162b2 second dose	1,301,148	86,334	6	11.9	0.50 (0.23 to 1.12)
Platelet disorder	ChAdOx1 first dose	3,760,221	277,527	380	247.4	1.54 (1.39 to 1.70)
Platelet disorder	ChAdOx1 second dose	1,088,684	57,845	111	78.3	1.42 (1.18 to 1.71)
Platelet disorder	BNT162b2 first dose	1,828,224	139,197	212	164.9	1.29 (1.12 to 1.47)
Platelet disorder	BNT162b2 second dose	1,298,763	86,169	141	114.8	1.23 (1.04 to 1.45)
Platelet disorder	SARS-CoV-2 PCR positive test	401,255	95,650	108	51	2.12 (1.75 to 2.56)
Portal vein thrombosis	ChAdOx1 first dose	3,764,456	277,862	14	11.2	1.24 (0.74 to 2.10)
Portal vein thrombosis	SARS-CoV-2 PCR positive test	401,566	95,736	8	2.3	3.51 (1.76 to 7.03)
Pulmonary embolism	ChAdOx1 first dose	3,757,618	277,328	466	370.2	1.26 (1.15 to 1.38)
Pulmonary embolism	ChAdOx1 second dose	1,087,821	57,800	99	124.8	0.79 (0.65 to 0.97)
Pulmonary embolism	BNT162b2 first dose	1,826,976	139,097	324	258.3	1.25 (1.12 to 1.40)
Pulmonary embolism	BNT162b2 second dose	1,298,128	86,131	153	182.7	0.84 (0.71 to 0.98)

Pulmonary embolism	SARS-CoV-2 PCR positive test	401,143	95,487	876	68.6	12.77 (11.95 to 13.64)
Pulmonary embolism with thrombocytopenia 10 days pre to 10 days post	ChAdOx1 first dose	3,764,563	277,870	8	4.6	1.72 (0.86 to 3.44)
Pulmonary embolism with thrombocytopenia 10 days pre to 10 days post	SARS-CoV-2 PCR positive test	401,571	95,737	9	0.8	11.95 (6.22 to 22.97)
Pulmonary embolism with thrombocytopenia 42 days pre to 14 days post	ChAdOx1 first dose	3,764,519	277,867	11	8.1	1.36 (0.75 to 2.45)
Pulmonary embolism with thrombocytopenia 42 days pre to 14 days post	BNT162b2 first dose	1,831,435	139,449	7	5.9	1.18 (0.56 to 2.47)
Pulmonary embolism with thrombocytopenia 42 days pre to 14 days post	BNT162b2 second dose	1,301,209	86,338	5	4.3	1.17 (0.49 to 2.82)
Pulmonary embolism with thrombocytopenia 42 days pre to 14 days post	SARS-CoV-2 PCR positive test	401,571	95,737	12	1.3	9.23 (5.24 to 16.25)
Splanchnic Vein Thrombosis	ChAdOx1 first dose	3,764,449	277,862	17	13.9	1.22 (0.76 to 1.97)
Splanchnic Vein Thrombosis	ChAdOx1 second dose	1,090,856	57,967	5	3.7	1.34 (0.56 to 3.21)
Splanchnic Vein Thrombosis	SARS-CoV-2 PCR positive test	401,564	95,736	8	2.8	2.81 (1.40 to 5.61)
Stroke	ChAdOx1 first dose	3,755,737	277,180	635	723.8	0.88 (0.81 to 0.95)
Stroke	ChAdOx1 second dose	1,086,113	57,695	216	287.1	0.75 (0.66 to 0.86)
Stroke	BNT162b2 first dose	1,824,739	138,920	537	613.3	0.88 (0.80 to 0.95)
Stroke	BNT162b2 second dose	1,296,430	86,003	312	460.4	0.68 (0.61 to 0.76)
Stroke	SARS-CoV-2 PCR positive test	401,062	95,608	121	111.3	1.09 (0.91 to 1.30)

Stroke with thrombocytopenia 10 days pre to 10 days post	ChAdOx1 first dose	3,764, 565	277,870	7	6.7	1.05 (0.50 to 2.21)
Stroke with thrombocytopenia 10 days pre to 10 days post	BNT162b2 first dose	1,831, 463	139,451	5	6.2	0.80 (0.33 to 1.93)
Stroke with thrombocytopenia 42 days pre to 14 days post	ChAdOx1 first dose	3,764, 529	277,868	7	11.9	0.59 (0.28 to 1.24)
Stroke with thrombocytopenia 42 days pre to 14 days post	ChAdOx1 second dose	1,090, 859	57,966	7	5.5	1.28 (0.61 to 2.68)
Stroke with thrombocytopenia 42 days pre to 14 days post	BNT162b2 first dose	1,831, 415	139,448	10	11.6	0.86 (0.46 to 1.60)
Thrombocytopenia	ChAdOx1 first dose	3,728, 941	275,077	2,615	1,824.10	1.43 (1.38 to 1.49)
Thrombocytopenia	ChAdOx1 second dose	1,070, 404	56,790	886	603	1.47 (1.38 to 1.57)
Thrombocytopenia	BNT162b2 first dose	1,801, 502	137,106	1,653	1,298.80	1.27 (1.21 to 1.34)
Thrombocytopenia	BNT162b2 second dose	1,277, 216	84,660	1,229	926.8	1.33 (1.25 to 1.40)
Thrombocytopenia	SARS-CoV-2 PCR positive test	399,23 9	95,143	536	384.8	1.39 (1.28 to 1.52)
Thrombocytopenic purpura	ChAdOx1 first dose	3,764, 491	277,864	31	14.6	2.13 (1.50 to 3.02)
Thrombocytopenic purpura	BNT162b2 first dose	1,831, 387	139,445	9	9.5	0.95 (0.49 to 1.82)
Thrombocytopenic purpura	SARS-CoV-2 PCR positive test	401,56 1	95,735	7	3.6	1.93 (0.92 to 4.04)
Venous thromboembolism - broad	ChAdOx1 first dose	3,750, 228	276,749	963	826.2	1.17 (1.09 to 1.24)
Venous thromboembolism - broad	ChAdOx1 second dose	1,084, 594	57,620	229	266.9	0.86 (0.75 to 0.98)

Venous thromboembolism - broad	BNT162b2 first dose	1,822,257	138,725	642	564.9	1.14 (1.05 to 1.23)
Venous thromboembolism - broad	BNT162b2 second dose	1,294,840	85,906	361	397.6	0.91 (0.82 to 1.01)
Venous thromboembolism - broad	SARS-CoV-2 PCR positive test	400,648	95,335	1,121	162.5	6.90 (6.51 to 7.31)
Venous thromboembolism - narrow	ChAdOx1 first dose	3,751,401	276,841	866	770.9	1.12 (1.05 to 1.20)
Venous thromboembolism - narrow	ChAdOx1 second dose	1,085,037	57,645	211	252	0.84 (0.73 to 0.96)
Venous thromboembolism - narrow	BNT162b2 first dose	1,822,927	138,779	595	533.2	1.12 (1.03 to 1.21)
Venous thromboembolism - narrow	BNT162b2 second dose	1,295,309	85,938	324	376.9	0.86 (0.77 to 0.96)
Venous thromboembolism - narrow	SARS-CoV-2 PCR positive test	400,723	95,357	1,090	149.8	7.27 (6.86 to 7.72)
Venous thromboembolism broad with thrombocytopenia 10 days pre to 10 days post	ChAdOx1 first dose	3,764,478	277,863	21	12	1.75 (1.14 to 2.68)
Venous thromboembolism broad with thrombocytopenia 10 days pre to 10 days post	BNT162b2 first dose	1,831,396	139,446	6	8.6	0.70 (0.31 to 1.56)
Venous thromboembolism broad with thrombocytopenia 10 days pre to 10 days post	BNT162b2 second dose	1,301,175	86,336	10	6.1	1.64 (0.88 to 3.05)
Venous thromboembolism broad with thrombocytopenia 10 days pre to 10 days post	SARS-CoV-2 PCR positive test	401,566	95,736	11	2	5.54 (3.07 to 10.00)
Venous thromboembolism broad with thrombocytopenia 42 days pre to 14 days post	ChAdOx1 first dose	3,764,398	277,857	29	19.4	1.50 (1.04 to 2.16)
Venous thromboembolism broad with thrombocytopenia 42 days pre to 14 days post	ChAdOx1 second dose	1,090,793	57,963	6	7	0.86 (0.39 to 1.92)
Venous thromboembolism broad with thrombocytopenia 42 days pre to 14 days post	BNT162b2 first dose	1,831,329	139,441	12	14.1	0.85 (0.48 to 1.50)

Venous thromboembolism broad with thrombocytopenia 42 days pre to 14 days post	BNT162b2 second dose	1,301,119	86,332	16	10.1	1.58 (0.97 to 2.58)
Venous thromboembolism broad with thrombocytopenia 42 days pre to 14 days post	SARS-CoV-2 PCR positive test	401,562	95,735	14	3.2	4.40 (2.61 to 7.43)
Venous thromboembolism narrow with thrombocytopenia 10 days pre to 10 days post	ChAdOx1 first dose	3,764,482	277,864	16	11.6	1.38 (0.85 to 2.26)
Venous thromboembolism narrow with thrombocytopenia 10 days pre to 10 days post	BNT162b2 first dose	1,831,401	139,447	6	8.1	0.74 (0.33 to 1.64)
Venous thromboembolism narrow with thrombocytopenia 10 days pre to 10 days post	BNT162b2 second dose	1,301,179	86,336	10	5.8	1.73 (0.93 to 3.22)
Venous thromboembolism narrow with thrombocytopenia 10 days pre to 10 days post	SARS-CoV-2 PCR positive test	401,566	95,736	11	1.9	5.76 (3.19 to 10.40)
Venous thromboembolism narrow with thrombocytopenia 42 days pre to 14 days post	ChAdOx1 first dose	3,764,403	277,858	24	18.6	1.29 (0.87 to 1.93)
Venous thromboembolism narrow with thrombocytopenia 42 days pre to 14 days post	ChAdOx1 second dose	1,090,796	57,963	5	6.7	0.75 (0.31 to 1.80)
Venous thromboembolism narrow with thrombocytopenia 42 days pre to 14 days post	BNT162b2 first dose	1,831,336	139,441	12	13.5	0.89 (0.50 to 1.56)
Venous thromboembolism narrow with thrombocytopenia 42 days pre to 14 days post	BNT162b2 second dose	1,301,125	86,332	15	9.7	1.55 (0.93 to 2.57)
Venous thromboembolism narrow with thrombocytopenia 42 days pre to 14 days post	SARS-CoV-2 PCR positive test	401,562	95,735	14	3	4.61 (2.73 to 7.78)

Supplementary Table 3. Results without requiring year of prior history

For each event of interest the number of persons contributing to the analysis from the target population, their person-years contributed, and the number of events observed for them are given. Their expected events are estimated using indirect standardisation to the general population, with expected events giving the number of events we would have expected to have seen if their outcome experience was the same as that of the general population. Standardised incidence ratios (SIRs) with 95% confidence intervals (CIs) were estimated. Events with less than 5 occurrences have been omitted for privacy reasons.

Event	Cohort	N	Person-years	Observed events	Expected events	SIR (95% CI)
Cerebral venous sinus thrombosis	Vaccinated with ChAdOx1 first dose	4,030,955	297,204	21	4.1	5.06 (3.30 to 7.77)
Cerebral venous sinus thrombosis	SARS-CoV-2 PCR positive test	437,899	104,181	5	1.4	3.49 (1.45 to 8.37)
Deep vein thrombosis - broad	Vaccinated with ChAdOx1 first dose	4,022,679	296,554	618	539.1	1.15 (1.06 to 1.24)
Deep vein thrombosis - broad	Vaccinated with ChAdOx1 second dose	1,142,233	60,886	152	166	0.92 (0.78 to 1.07)
Deep vein thrombosis - broad	Vaccinated with BNT162b2 first dose	1,945,899	148,061	375	355.1	1.06 (0.95 to 1.17)
Deep vein thrombosis - broad	Vaccinated with BNT162b2 second dose	1,364,360	90,615	235	245.6	0.96 (0.84 to 1.09)
Deep vein thrombosis - broad	SARS-CoV-2 PCR positive test	437,333	104,002	326	112.3	2.90 (2.61 to 3.24)
Deep vein thrombosis - narrow	Vaccinated with ChAdOx1 first dose	4,023,889	296,650	512	479	1.07 (0.98 to 1.17)
Deep vein thrombosis - narrow	Vaccinated with ChAdOx1 second dose	1,142,686	60,912	133	149.9	0.89 (0.75 to 1.05)
Deep vein thrombosis - narrow	Vaccinated with BNT162b2 first dose	1,946,579	148,115	323	321	1.01 (0.90 to 1.12)
Deep vein thrombosis - narrow	Vaccinated with BNT162b2 second dose	1,364,834	90,647	194	223.5	0.87 (0.75 to 1.00)

Deep vein thrombosis - narrow	SARS-CoV-2 PCR positive test	437,412	104,025	290	98.3	2.95 (2.63 to 3.31)
Deep vein thrombosis broad with thrombocytopenia 10 days pre to 10 days post	Vaccinated with ChAdOx1 first dose	4,030,995	297,207	20	8.4	2.39 (1.54 to 3.71)
Deep vein thrombosis broad with thrombocytopenia 10 days pre to 10 days post	Vaccinated with ChAdOx1 second dose	1,145,817	61,087	5	2.8	1.78 (0.74 to 4.27)
Deep vein thrombosis broad with thrombocytopenia 10 days pre to 10 days post	Vaccinated with BNT162b2 second dose	1,367,937	90,859	8	4	1.99 (0.99 to 3.97)
Deep vein thrombosis broad with thrombocytopenia 42 days pre to 14 days post	Vaccinated with ChAdOx1 first dose	4,030,955	297,204	25	13.1	1.91 (1.29 to 2.83)
Deep vein thrombosis broad with thrombocytopenia 42 days pre to 14 days post	Vaccinated with ChAdOx1 second dose	1,145,784	61,085	6	4.6	1.30 (0.58 to 2.90)
Deep vein thrombosis broad with thrombocytopenia 42 days pre to 14 days post	Vaccinated with BNT162b2 first dose	1,951,049	148,468	8	9.3	0.86 (0.43 to 1.72)
Deep vein thrombosis broad with thrombocytopenia 42 days pre to 14 days post	Vaccinated with BNT162b2 second dose	1,367,902	90,856	12	6.6	1.82 (1.03 to 3.20)
Deep vein thrombosis narrow with thrombocytopenia 10 days pre to 10 days post	Vaccinated with ChAdOx1 first dose	4,031,002	297,207	14	7.8	1.80 (1.06 to 3.03)
Deep vein thrombosis narrow with thrombocytopenia 10 days pre to 10 days post	Vaccinated with BNT162b2 second dose	1,367,942	90,859	8	3.7	2.16 (1.08 to 4.32)
Deep vein thrombosis narrow with thrombocytopenia 42 days pre to 14 days post	Vaccinated with ChAdOx1 first dose	4,030,964	297,204	19	12.2	1.56 (0.99 to 2.44)
Deep vein thrombosis narrow with thrombocytopenia 42 days pre to 14 days post	Vaccinated with ChAdOx1 second dose	1,145,791	61,085	5	4.3	1.16 (0.48 to 2.78)
Deep vein thrombosis narrow with thrombocytopenia 42 days pre to 14 days post	Vaccinated with BNT162b2 first dose	1,951,057	148,468	8	8.7	0.92 (0.46 to 1.84)
Deep vein thrombosis narrow with thrombocytopenia 42 days pre to 14 days post	Vaccinated with BNT162b2 second dose	1,367,909	90,857	11	6.2	1.79 (0.99 to 3.23)
Hemorrhagic stroke	Vaccinated with ChAdOx1 first dose	4,029,750	297,111	88	97.2	0.91 (0.73 to 1.12)

Hemorrhagic stroke	Vaccinated with ChAdOx1 second dose	1,145, 341	61,059	24	32.2	0.75 (0.50 to 1.11)
Hemorrhagic stroke	Vaccinated with BNT162b2 first dose	1,950, 465	148,422	45	70.8	0.64 (0.47 to 0.85)
Hemorrhagic stroke	Vaccinated with BNT162b2 second dose	1,367, 548	90,832	30	50.7	0.59 (0.41 to 0.85)
Hemorrhagic stroke	SARS-CoV-2 PCR positive test	437,7 97	104,155	24	19.5	1.23 (0.82 to 1.84)
Immune thrombocytopenia	Vaccinated with ChAdOx1 first dose	4,030, 643	297,179	49	26.9	1.82 (1.37 to 2.41)
Immune thrombocytopenia	Vaccinated with ChAdOx1 second dose	1,145, 650	61,077	8	8.2	0.98 (0.49 to 1.96)
Immune thrombocytopenia	Vaccinated with BNT162b2 first dose	1,950, 837	148,451	22	17.3	1.27 (0.84 to 1.93)
Immune thrombocytopenia	Vaccinated with BNT162b2 second dose	1,367, 755	90,847	10	11.8	0.84 (0.45 to 1.57)
Immune thrombocytopenia	SARS-CoV-2 PCR positive test	437,8 69	104,174	10	6.4	1.56 (0.84 to 2.91)
Intestinal infarction	Vaccinated with ChAdOx1 first dose	4,030, 668	297,182	31	25.6	1.21 (0.85 to 1.72)
Intestinal infarction	Vaccinated with ChAdOx1 second dose	1,145, 663	61,078	5	8.7	0.57 (0.24 to 1.38)
Intestinal infarction	Vaccinated with BNT162b2 first dose	1,950, 906	148,456	19	17.8	1.07 (0.68 to 1.67)
Intestinal infarction	Vaccinated with BNT162b2 second dose	1,367, 813	90,851	10	12.5	0.80 (0.43 to 1.48)
Intestinal infarction	SARS-CoV-2 PCR positive test	437,8 78	104,175	8	4.5	1.76 (0.88 to 3.52)
Ischemic stroke	Vaccinated with ChAdOx1 first dose	4,028, 946	297,047	145	167	0.87 (0.74 to 1.02)

Ischemic stroke	Vaccinated with ChAdOx1 second dose	1,144,754	61,027	53	67.4	0.79 (0.60 to 1.03)
Ischemic stroke	Vaccinated with BNT162b2 first dose	1,949,598	148,352	154	140.2	1.10 (0.94 to 1.29)
Ischemic stroke	Vaccinated with BNT162b2 second dose	1,366,855	90,782	70	104.4	0.67 (0.53 to 0.85)
Ischemic stroke	SARS-CoV-2 PCR positive test	437,793	104,154	32	25.2	1.27 (0.90 to 1.79)
Mesenteric vein thrombosis	Vaccinated with ChAdOx1 first dose	4,031,032	297,210	7	3.6	1.92 (0.92 to 4.04)
Myocardial infarction	Vaccinated with ChAdOx1 first dose	4,021,775	296,482	657	752.7	0.87 (0.81 to 0.94)
Myocardial infarction	Vaccinated with ChAdOx1 second dose	1,141,173	60,833	176	250.5	0.70 (0.61 to 0.81)
Myocardial infarction	Vaccinated with BNT162b2 first dose	1,943,672	147,888	465	523	0.89 (0.81 to 0.97)
Myocardial infarction	Vaccinated with BNT162b2 second dose	1,363,163	90,537	301	368	0.82 (0.73 to 0.92)
Myocardial infarction	SARS-CoV-2 PCR positive test	437,282	104,016	172	122.2	1.41 (1.21 to 1.63)
Myocardial infarction or ischemic stroke	Vaccinated with ChAdOx1 first dose	4,018,772	296,250	779	893.8	0.87 (0.81 to 0.94)
Myocardial infarction or ischemic stroke	Vaccinated with ChAdOx1 second dose	1,139,688	60,750	225	309.2	0.73 (0.64 to 0.83)
Myocardial infarction or ischemic stroke	Vaccinated with BNT162b2 first dose	1,941,398	147,710	599	645.5	0.93 (0.86 to 1.01)
Myocardial infarction or ischemic stroke	Vaccinated with BNT162b2 second dose	1,361,610	90,429	364	460.1	0.79 (0.71 to 0.88)
Myocardial infarction or ischemic stroke	SARS-CoV-2 PCR positive test	437,111	103,973	195	143.3	1.36 (1.18 to 1.57)

Myocardial infarction or ischemic stroke with thrombocytopenia 10 days pre to 10 days post	Vaccinated with ChAdOx1 first dose	4,030,988	297,207	7	12.1	0.58 (0.28 to 1.21)
Myocardial infarction or ischemic stroke with thrombocytopenia 10 days pre to 10 days post	Vaccinated with BNT162b2 first dose	1,951,059	148,469	7	10.2	0.69 (0.33 to 1.44)
Myocardial infarction or ischemic stroke with thrombocytopenia 42 days pre to 14 days post	Vaccinated with ChAdOx1 first dose	4,030,922	297,201	12	19.7	0.61 (0.35 to 1.07)
Myocardial infarction or ischemic stroke with thrombocytopenia 42 days pre to 14 days post	Vaccinated with BNT162b2 first dose	1,951,009	148,464	12	16.5	0.73 (0.41 to 1.28)
Myocardial infarction or ischemic stroke with thrombocytopenia 42 days pre to 14 days post	Vaccinated with BNT162b2 second dose	1,367,873	90,854	6	12.3	0.49 (0.22 to 1.09)
Platelet disorder	Vaccinated with ChAdOx1 first dose	4,026,496	296,851	419	263.9	1.59 (1.44 to 1.75)
Platelet disorder	Vaccinated with ChAdOx1 second dose	1,143,542	60,958	117	82.5	1.42 (1.18 to 1.70)
Platelet disorder	Vaccinated with BNT162b2 first dose	1,947,779	148,211	225	173.2	1.30 (1.14 to 1.48)
Platelet disorder	Vaccinated with BNT162b2 second dose	1,365,428	90,686	146	119.2	1.22 (1.04 to 1.44)
Platelet disorder	SARS-CoV-2 PCR positive test	437,567	104,088	119	55.4	2.15 (1.79 to 2.57)
Portal vein thrombosis	Vaccinated with ChAdOx1 first dose	4,030,905	297,200	15	11.9	1.26 (0.76 to 2.09)
Portal vein thrombosis	SARS-CoV-2 PCR positive test	437,899	104,181	8	2.4	3.32 (1.66 to 6.63)
Pulmonary embolism	Vaccinated with ChAdOx1 first dose	4,023,712	296,638	510	393.8	1.29 (1.19 to 1.41)
Pulmonary embolism	Vaccinated with ChAdOx1 second dose	1,142,618	60,910	106	131.7	0.81 (0.67 to 0.97)
Pulmonary embolism	Vaccinated with BNT162b2 first dose	1,946,444	148,104	352	271.4	1.30 (1.17 to 1.44)

Pulmonary embolism	Vaccinated with BNT162b2 second dose	1,364,743	90,643	169	190.2	0.89 (0.76 to 1.03)
Pulmonary embolism	SARS-CoV-2 PCR positive test	437,439	103,916	919	73.7	12.47 (11.68 to 13.30)
Pulmonary embolism with thrombocytopenia 10 days pre to 10 days post	Vaccinated with ChAdOx1 first dose	4,031,021	297,209	9	4.9	1.83 (0.95 to 3.51)
Pulmonary embolism with thrombocytopenia 10 days pre to 10 days post	SARS-CoV-2 PCR positive test	437,904	104,182	9	0.8	11.17 (5.81 to 21.46)
Pulmonary embolism with thrombocytopenia 42 days pre to 14 days post	Vaccinated with ChAdOx1 first dose	4,030,975	297,206	12	8.7	1.38 (0.79 to 2.43)
Pulmonary embolism with thrombocytopenia 42 days pre to 14 days post	Vaccinated with BNT162b2 first dose	1,951,086	148,471	7	6.3	1.12 (0.53 to 2.35)
Pulmonary embolism with thrombocytopenia 42 days pre to 14 days post	Vaccinated with BNT162b2 second dose	1,367,935	90,859	5	4.4	1.13 (0.47 to 2.70)
Pulmonary embolism with thrombocytopenia 42 days pre to 14 days post	SARS-CoV-2 PCR positive test	437,904	104,181	13	1.4	9.31 (5.41 to 16.03)
Splanchnic Vein Thrombosis	Vaccinated with ChAdOx1 first dose	4,030,894	297,199	20	14.8	1.35 (0.87 to 2.10)
Splanchnic Vein Thrombosis	Vaccinated with ChAdOx1 second dose	1,145,795	61,085	6	3.9	1.52 (0.68 to 3.39)
Splanchnic Vein Thrombosis	Vaccinated with BNT162b2 first dose	1,951,055	148,468	5	8.1	0.62 (0.26 to 1.48)
Splanchnic Vein Thrombosis	SARS-CoV-2 PCR positive test	437,897	104,180	9	3	2.97 (1.54 to 5.70)
Stroke	Vaccinated with ChAdOx1 first dose	4,021,552	296,467	746	776.8	0.96 (0.89 to 1.03)
Stroke	Vaccinated with ChAdOx1 second dose	1,140,713	60,792	248	306.3	0.81 (0.71 to 0.92)
Stroke	Vaccinated with BNT162b2 first dose	1,944,022	147,912	573	648.6	0.88 (0.81 to 0.96)

Stroke	Vaccinated with BNT162b2 second dose	1,362,922	90,507	329	482.5	0.68 (0.61 to 0.76)
Stroke	SARS-CoV-2 PCR positive test	437,336	104,039	145	121.5	1.19 (1.01 to 1.40)
Stroke with thrombocytopenia 10 days pre to 10 days post	Vaccinated with ChAdOx1 first dose	4,031,018	297,209	7	7.2	0.97 (0.46 to 2.04)
Stroke with thrombocytopenia 10 days pre to 10 days post	Vaccinated with BNT162b2 first dose	1,951,114	148,473	5	6.6	0.76 (0.32 to 1.83)
Stroke with thrombocytopenia 42 days pre to 14 days post	Vaccinated with ChAdOx1 first dose	4,030,982	297,206	9	12.9	0.70 (0.36 to 1.34)
Stroke with thrombocytopenia 42 days pre to 14 days post	Vaccinated with ChAdOx1 second dose	1,145,800	61,085	7	5.8	1.20 (0.57 to 2.51)
Stroke with thrombocytopenia 42 days pre to 14 days post	Vaccinated with BNT162b2 first dose	1,951,064	148,469	10	12.2	0.82 (0.44 to 1.52)
Thrombocytopenia	Vaccinated with ChAdOx1 first dose	3,994,045	294,304	2,900	1,956.50	1.48 (1.43 to 1.54)
Thrombocytopenia	Vaccinated with ChAdOx1 second dose	1,124,655	59,864	959	639.4	1.50 (1.41 to 1.60)
Thrombocytopenia	Vaccinated with BNT162b2 first dose	1,920,359	146,063	1,769	1,371.40	1.29 (1.23 to 1.35)
Thrombocytopenia	Vaccinated with BNT162b2 second dose	1,343,405	89,142	1,296	967.8	1.34 (1.27 to 1.41)
Thrombocytopenia	SARS-CoV-2 PCR positive test	435,427	103,549	598	420.8	1.42 (1.31 to 1.54)
Thrombocytopenic purpura	Vaccinated with ChAdOx1 first dose	4,030,933	297,202	35	15.9	2.20 (1.58 to 3.06)
Thrombocytopenic purpura	Vaccinated with BNT162b2 first dose	1,951,028	148,466	10	10.2	0.98 (0.53 to 1.82)
Thrombocytopenic purpura	Vaccinated with BNT162b2 second dose	1,367,907	90,857	5	7	0.72 (0.30 to 1.72)

Thrombocytopenic purpura	SARS-CoV-2 PCR positive test	437,891	104,179	7	4.1	1.71 (0.82 to 3.60)
Venous thromboembolism - broad	Vaccinated with ChAdOx1 first dose	4,015,942	296,028	1,064	880.3	1.21 (1.14 to 1.28)
Venous thromboembolism - broad	Vaccinated with ChAdOx1 second dose	1,139,239	60,720	249	282.4	0.88 (0.78 to 1.00)
Venous thromboembolism - broad	Vaccinated with BNT162b2 first dose	1,941,550	147,718	692	594.9	1.16 (1.08 to 1.25)
Venous thromboembolism - broad	Vaccinated with BNT162b2 second dose	1,361,347	90,410	392	414.8	0.94 (0.86 to 1.04)
Venous thromboembolism - broad	SARS-CoV-2 PCR positive test	436,911	103,753	1,189	174.9	6.80 (6.42 to 7.19)
Venous thromboembolism - narrow	Vaccinated with ChAdOx1 first dose	4,017,147	296,123	960	822	1.17 (1.10 to 1.24)
Venous thromboembolism - narrow	Vaccinated with ChAdOx1 second dose	1,139,697	60,746	230	266.9	0.86 (0.76 to 0.98)
Venous thromboembolism - narrow	Vaccinated with BNT162b2 first dose	1,942,237	147,772	643	561.9	1.14 (1.06 to 1.24)
Venous thromboembolism - narrow	Vaccinated with BNT162b2 second dose	1,361,822	90,443	352	393.4	0.89 (0.81 to 0.99)
Venous thromboembolism - narrow	SARS-CoV-2 PCR positive test	436,989	103,776	1,157	161.4	7.17 (6.77 to 7.59)
Venous thromboembolism broad with thrombocytopenia 10 days pre to 10 days post	Vaccinated with ChAdOx1 first dose	4,030,931	297,202	25	12.8	1.96 (1.32 to 2.90)
Venous thromboembolism broad with thrombocytopenia 10 days pre to 10 days post	Vaccinated with ChAdOx1 second dose	1,145,784	61,085	6	4.4	1.36 (0.61 to 3.04)
Venous thromboembolism broad with thrombocytopenia 10 days pre to 10 days post	Vaccinated with BNT162b2 first dose	1,951,045	148,467	7	8.9	0.78 (0.37 to 1.64)
Venous thromboembolism broad with thrombocytopenia 10 days pre to 10 days post	Vaccinated with BNT162b2 second dose	1,367,899	90,856	10	6.3	1.59 (0.86 to 2.96)

Venous thromboembolism broad with thrombocytopenia 10 days pre to 10 days post	SARS-CoV-2 PCR positive test	437,899	104,181	11	2.1	5.17 (2.86 to 9.34)
Venous thromboembolism broad with thrombocytopenia 42 days pre to 14 days post	Vaccinated with ChAdOx1 first dose	4,030,848	297,195	33	20.7	1.60 (1.14 to 2.25)
Venous thromboembolism broad with thrombocytopenia 42 days pre to 14 days post	Vaccinated with ChAdOx1 second dose	1,145,727	61,082	8	7.4	1.09 (0.54 to 2.17)
Venous thromboembolism broad with thrombocytopenia 42 days pre to 14 days post	Vaccinated with BNT162b2 first dose	1,950,977	148,462	13	14.8	0.88 (0.51 to 1.51)
Venous thromboembolism broad with thrombocytopenia 42 days pre to 14 days post	Vaccinated with BNT162b2 second dose	1,367,843	90,852	16	10.5	1.52 (0.93 to 2.48)
Venous thromboembolism broad with thrombocytopenia 42 days pre to 14 days post	SARS-CoV-2 PCR positive test	437,895	104,179	15	3.4	4.38 (2.64 to 7.27)
Venous thromboembolism narrow with thrombocytopenia 10 days pre to 10 days post	Vaccinated with ChAdOx1 first dose	4,030,937	297,203	19	12.2	1.56 (0.99 to 2.44)
Venous thromboembolism narrow with thrombocytopenia 10 days pre to 10 days post	Vaccinated with ChAdOx1 second dose	1,145,788	61,085	5	4.2	1.19 (0.50 to 2.86)
Venous thromboembolism narrow with thrombocytopenia 10 days pre to 10 days post	Vaccinated with BNT162b2 first dose	1,951,050	148,468	7	8.5	0.83 (0.39 to 1.73)
Venous thromboembolism narrow with thrombocytopenia 10 days pre to 10 days post	Vaccinated with BNT162b2 second dose	1,367,903	90,856	10	5.9	1.68 (0.91 to 3.13)
Venous thromboembolism narrow with thrombocytopenia 10 days pre to 10 days post	SARS-CoV-2 PCR positive test	437,899	104,181	11	2	5.41 (2.99 to 9.76)
Venous thromboembolism narrow with thrombocytopenia 42 days pre to 14 days post	Vaccinated with ChAdOx1 first dose	4,030,855	297,196	27	19.8	1.36 (0.93 to 1.99)
Venous thromboembolism narrow with thrombocytopenia 42 days pre to 14 days post	Vaccinated with ChAdOx1 second dose	1,145,732	61,082	7	7.1	0.99 (0.47 to 2.08)
Venous thromboembolism narrow with thrombocytopenia 42 days pre to 14 days post	Vaccinated with BNT162b2 first dose	1,950,984	148,463	13	14.2	0.91 (0.53 to 1.58)
Venous thromboembolism narrow with thrombocytopenia 42 days pre to 14 days post	Vaccinated with BNT162b2 second dose	1,367,849	90,853	15	10.1	1.49 (0.90 to 2.47)

Venous thromboembolism narrow with thrombocytopenia 42 days pre to 14 days post	SARS-CoV-2 PCR positive test	437,8 95	104,179	15	3.3	4.60 (2.77 to 7.63)

Supplementary Table 4. Results with general population identified based on a visit/ contact

For each event of interest the number of persons contributing to the analysis from the target population, their person-years contributed, and the number of events observed for them are given. Their expected events are estimated using indirect standardisation to the general population, with expected events giving the number of events we would have expected to have seen if their outcome experience was the same as that of the general population. Standardised incidence ratios (SIRs) with 95% confidence intervals (CIs) were estimated. Events with less than 5 occurrences have been omitted for privacy reasons.

Event	Cohort	N	Person-years	Observed events	Expected events	SIR (95% CI)
Cerebral venous sinus thrombosis	Vaccinated with ChAdOx1 first dose	3,764,507	277,866	16	4.2	3.78 (2.32 to 6.17)
Cerebral venous sinus thrombosis	SARS-CoV-2 PCR positive test	401,568	95,737	5	1.5	3.26 (1.36 to 7.84)
Deep vein thrombosis - broad	Vaccinated with ChAdOx1 first dose	3,756,629	277,248	555	538	1.03 (0.95 to 1.12)
Deep vein thrombosis - broad	Vaccinated with ChAdOx1 second dose	1,087,452	57,778	139	163.5	0.85 (0.72 to 1.00)
Deep vein thrombosis - broad	Vaccinated with BNT162b2 first dose	1,826,442	139,055	353	353.7	1.00 (0.90 to 1.11)
Deep vein thrombosis - broad	Vaccinated with BNT162b2 second dose	1,297,754	86,102	220	246.1	0.89 (0.78 to 1.02)
Deep vein thrombosis - broad	SARS-CoV-2 PCR positive test	401,035	95,569	299	114.5	2.61 (2.33 to 2.93)
Deep vein thrombosis - narrow	Vaccinated with ChAdOx1 first dose	3,757,806	277,341	456	477.5	0.95 (0.87 to 1.05)
Deep vein thrombosis - narrow	Vaccinated with ChAdOx1 second dose	1,087,890	57,802	121	147.5	0.82 (0.69 to 0.98)
Deep vein thrombosis - narrow	Vaccinated with BNT162b2 first dose	1,827,104	139,108	303	319.4	0.95 (0.85 to 1.06)
Deep vein thrombosis - narrow	Vaccinated with BNT162b2 second dose	1,298,221	86,134	182	223.7	0.81 (0.70 to 0.94)

Deep vein thrombosis - narrow	SARS-CoV-2 PCR positive test	401,111	95,592	265	100.1	2.65 (2.35 to 2.99)
Deep vein thrombosis broad with thrombocytopenia 10 days pre to 10 days post	Vaccinated with ChAdOx1 first dose	3,764,541	277,868	16	8.3	1.93 (1.18 to 3.15)
Deep vein thrombosis broad with thrombocytopenia 10 days pre to 10 days post	Vaccinated with BNT162b2 second dose	1,301,213	86,338	8	4.1	1.97 (0.98 to 3.94)
Deep vein thrombosis broad with thrombocytopenia 42 days pre to 14 days post	Vaccinated with ChAdOx1 first dose	3,764,502	277,865	21	12.9	1.63 (1.06 to 2.50)
Deep vein thrombosis broad with thrombocytopenia 42 days pre to 14 days post	Vaccinated with BNT162b2 first dose	1,831,401	139,447	7	9.3	0.75 (0.36 to 1.58)
Deep vein thrombosis broad with thrombocytopenia 42 days pre to 14 days post	Vaccinated with BNT162b2 second dose	1,301,178	86,336	12	6.6	1.81 (1.03 to 3.19)
Deep vein thrombosis narrow with thrombocytopenia 10 days pre to 10 days post	Vaccinated with ChAdOx1 first dose	3,764,546	277,869	11	7.7	1.42 (0.79 to 2.56)
Deep vein thrombosis narrow with thrombocytopenia 10 days pre to 10 days post	Vaccinated with BNT162b2 second dose	1,301,218	86,339	8	3.7	2.14 (1.07 to 4.29)
Deep vein thrombosis narrow with thrombocytopenia 42 days pre to 14 days post	Vaccinated with ChAdOx1 first dose	3,764,509	277,866	16	12	1.33 (0.81 to 2.17)
Deep vein thrombosis narrow with thrombocytopenia 42 days pre to 14 days post	Vaccinated with BNT162b2 first dose	1,831,409	139,447	7	8.7	0.81 (0.39 to 1.70)
Deep vein thrombosis narrow with thrombocytopenia 42 days pre to 14 days post	Vaccinated with BNT162b2 second dose	1,301,185	86,336	11	6.2	1.78 (0.98 to 3.21)
Hemorrhagic stroke	Vaccinated with ChAdOx1 first dose	3,763,357	277,777	74	95.1	0.78 (0.62 to 0.98)
Hemorrhagic stroke	Vaccinated with ChAdOx1 second dose	1,090,433	57,943	20	31.4	0.64 (0.41 to 0.99)
Hemorrhagic stroke	Vaccinated with BNT162b2 first dose	1,830,843	139,403	45	69.7	0.65 (0.48 to 0.86)
Hemorrhagic stroke	Vaccinated with BNT162b2 second dose	1,300,839	86,313	29	50.4	0.58 (0.40 to 0.83)

Hemorrhagic stroke	SARS-CoV-2 PCR positive test	401,471	95,713	20	19.4	1.03 (0.67 to 1.60)
Immune thrombocytopenia	Vaccinated with ChAdOx1 first dose	3,764,198	277,841	45	27	1.67 (1.25 to 2.24)
Immune thrombocytopenia	Vaccinated with ChAdOx1 second dose	1,090,713	57,959	8	8.1	0.99 (0.50 to 1.99)
Immune thrombocytopenia	Vaccinated with BNT162b2 first dose	1,831,194	139,430	21	17.3	1.21 (0.79 to 1.86)
Immune thrombocytopenia	Vaccinated with BNT162b2 second dose	1,301,033	86,326	9	11.9	0.75 (0.39 to 1.45)
Immune thrombocytopenia	SARS-CoV-2 PCR positive test	401,539	95,729	10	6.6	1.51 (0.81 to 2.80)
Intestinal infarction	Vaccinated with ChAdOx1 first dose	3,764,223	277,844	25	25.6	0.98 (0.66 to 1.44)
Intestinal infarction	Vaccinated with ChAdOx1 second dose	1,090,726	57,959	5	8.6	0.58 (0.24 to 1.39)
Intestinal infarction	Vaccinated with BNT162b2 first dose	1,831,256	139,435	17	17.8	0.96 (0.59 to 1.54)
Intestinal infarction	Vaccinated with BNT162b2 second dose	1,301,091	86,331	9	12.6	0.72 (0.37 to 1.37)
Intestinal infarction	SARS-CoV-2 PCR positive test	401,547	95,732	7	4.6	1.52 (0.73 to 3.20)
Ischemic stroke	Vaccinated with ChAdOx1 first dose	3,762,624	277,719	128	163.3	0.78 (0.66 to 0.93)
Ischemic stroke	Vaccinated with ChAdOx1 second dose	1,089,880	57,912	47	65.7	0.72 (0.54 to 0.95)
Ischemic stroke	Vaccinated with BNT162b2 first dose	1,830,001	139,335	146	138	1.06 (0.90 to 1.24)
Ischemic stroke	Vaccinated with BNT162b2 second dose	1,300,162	86,264	68	103.6	0.66 (0.52 to 0.83)

Ischemic stroke	SARS-CoV-2 PCR positive test	401,463	95,710	28	24.7	1.13 (0.78 to 1.64)
Major adverse cardiac events	Vaccinated with ChAdOx1 first dose	3,737,214	275,726	1,910	1,869.30	1.02 (0.98 to 1.07)
Major adverse cardiac events	Vaccinated with ChAdOx1 second dose	1,074,751	57,038	671	759.5	0.88 (0.82 to 0.95)
Major adverse cardiac events	Vaccinated with BNT162b2 first dose	1,806,857	137,520	1,576	1,651.70	0.95 (0.91 to 1.00)
Major adverse cardiac events	Vaccinated with BNT162b2 second dose	1,282,670	85,023	1,151	1,254.30	0.92 (0.87 to 0.97)
Major adverse cardiac events	SARS-CoV-2 PCR positive test	399,976	95,331	375	281.2	1.33 (1.21 to 1.48)
Major adverse cardiac events with thrombocytopenia 10 days pre to 10 days post	Vaccinated with ChAdOx1 first dose	3,764,097	277,833	54	54.3	0.99 (0.76 to 1.30)
Major adverse cardiac events with thrombocytopenia 10 days pre to 10 days post	Vaccinated with ChAdOx1 second dose	1,090,543	57,947	24	27.8	0.86 (0.58 to 1.29)
Major adverse cardiac events with thrombocytopenia 10 days pre to 10 days post	Vaccinated with BNT162b2 first dose	1,830,851	139,404	45	61.8	0.73 (0.54 to 0.98)
Major adverse cardiac events with thrombocytopenia 10 days pre to 10 days post	Vaccinated with BNT162b2 second dose	1,300,730	86,303	45	49.6	0.91 (0.68 to 1.22)
Major adverse cardiac events with thrombocytopenia 42 days pre to 14 days post	Vaccinated with ChAdOx1 first dose	3,763,784	277,809	75	84.3	0.89 (0.71 to 1.12)
Major adverse cardiac events with thrombocytopenia 42 days pre to 14 days post	Vaccinated with ChAdOx1 second dose	1,090,337	57,934	29	42.5	0.68 (0.47 to 0.98)
Major adverse cardiac events with thrombocytopenia 42 days pre to 14 days post	Vaccinated with BNT162b2 first dose	1,830,512	139,377	64	93.5	0.68 (0.54 to 0.87)
Major adverse cardiac events with thrombocytopenia 42 days pre to 14 days post	Vaccinated with BNT162b2 second dose	1,300,432	86,280	72	74.7	0.96 (0.76 to 1.21)
Major adverse cardiac events with thrombocytopenia 42 days pre to 14 days post	SARS-CoV-2 PCR positive test	401,527	95,728	8	10.6	0.75 (0.38 to 1.51)

Mesenteric vein thrombosis	Vaccinated with ChAdOx1 first dose	3,764,576	277,871	5	3.6	1.37 (0.57 to 3.30)
Myocardial infarction	Vaccinated with ChAdOx1 first dose	3,755,717	277,176	606	744.2	0.81 (0.75 to 0.88)
Myocardial infarction	Vaccinated with ChAdOx1 second dose	1,086,394	57,724	166	247.2	0.67 (0.58 to 0.78)
Myocardial infarction	Vaccinated with BNT162b2 first dose	1,824,272	138,887	442	520.7	0.85 (0.77 to 0.93)
Myocardial infarction	Vaccinated with BNT162b2 second dose	1,296,561	86,025	283	369.2	0.77 (0.68 to 0.86)
Myocardial infarction	SARS-CoV-2 PCR positive test	400,986	95,580	167	121.5	1.37 (1.18 to 1.60)
Myocardial infarction or ischemic stroke	Vaccinated with ChAdOx1 first dose	3,752,849	276,954	712	880.7	0.81 (0.75 to 0.87)
Myocardial infarction or ischemic stroke	Vaccinated with ChAdOx1 second dose	1,084,976	57,646	209	304	0.69 (0.60 to 0.79)
Myocardial infarction or ischemic stroke	Vaccinated with BNT162b2 first dose	1,822,051	138,713	568	640.5	0.89 (0.82 to 0.96)
Myocardial infarction or ischemic stroke	Vaccinated with BNT162b2 second dose	1,295,041	85,920	344	460.1	0.75 (0.67 to 0.83)
Myocardial infarction or ischemic stroke	SARS-CoV-2 PCR positive test	400,818	95,538	186	141.9	1.31 (1.14 to 1.51)
Myocardial infarction or ischemic stroke with thrombocytopenia 10 days pre to 10 days post	Vaccinated with ChAdOx1 first dose	3,764,535	277,868	7	12.2	0.58 (0.27 to 1.21)
Myocardial infarction or ischemic stroke with thrombocytopenia 10 days pre to 10 days post	Vaccinated with BNT162b2 first dose	1,831,410	139,447	6	10.3	0.58 (0.26 to 1.30)
Myocardial infarction or ischemic stroke with thrombocytopenia 42 days pre to 14 days post	Vaccinated with ChAdOx1 first dose	3,764,471	277,863	12	19.6	0.61 (0.35 to 1.08)
Myocardial infarction or ischemic stroke with thrombocytopenia 42 days pre to 14 days post	Vaccinated with BNT162b2 first dose	1,831,361	139,443	11	16.6	0.66 (0.37 to 1.20)

Myocardial infarction or ischemic stroke with thrombocytopenia 42 days pre to 14 days post	Vaccinated with BNT162b2 second dose	1,301,148	86,334	6	12.4	0.48 (0.22 to 1.07)
Platelet disorder	Vaccinated with ChAdOx1 first dose	3,760,221	277,527	380	264.2	1.44 (1.30 to 1.59)
Platelet disorder	Vaccinated with ChAdOx1 second dose	1,088,684	57,845	111	81.9	1.36 (1.13 to 1.63)
Platelet disorder	Vaccinated with BNT162b2 first dose	1,828,224	139,197	212	173.6	1.22 (1.07 to 1.40)
Platelet disorder	Vaccinated with BNT162b2 second dose	1,298,763	86,169	141	120.4	1.17 (0.99 to 1.38)
Platelet disorder	SARS-CoV-2 PCR positive test	401,255	95,650	108	56.6	1.91 (1.58 to 2.31)
Portal vein thrombosis	Vaccinated with ChAdOx1 first dose	3,764,456	277,862	14	12.1	1.16 (0.69 to 1.96)
Portal vein thrombosis	SARS-CoV-2 PCR positive test	401,566	95,736	8	2.5	3.20 (1.60 to 6.40)
Pulmonary embolism	Vaccinated with ChAdOx1 first dose	3,757,618	277,328	466	392.9	1.19 (1.08 to 1.30)
Pulmonary embolism	Vaccinated with ChAdOx1 second dose	1,087,821	57,800	99	130.3	0.76 (0.62 to 0.93)
Pulmonary embolism	Vaccinated with BNT162b2 first dose	1,826,976	139,097	324	271.1	1.19 (1.07 to 1.33)
Pulmonary embolism	Vaccinated with BNT162b2 second dose	1,298,128	86,131	153	191.2	0.80 (0.68 to 0.94)
Pulmonary embolism	SARS-CoV-2 PCR positive test	401,143	95,487	876	75	11.68 (10.93 to 12.48)
Pulmonary embolism with thrombocytopenia 10 days pre to 10 days post	Vaccinated with ChAdOx1 first dose	3,764,563	277,870	8	4.9	1.63 (0.81 to 3.26)
Pulmonary embolism with thrombocytopenia 10 days pre to 10 days post	SARS-CoV-2 PCR positive test	401,571	95,737	9	0.8	10.94 (5.69 to 21.03)

Pulmonary embolism with thrombocytopenia 42 days pre to 14 days post	Vaccinated with ChAdOx1 first dose	3,764,519	277,867	11	8.6	1.28 (0.71 to 2.32)
Pulmonary embolism with thrombocytopenia 42 days pre to 14 days post	Vaccinated with BNT162b2 first dose	1,831,435	139,449	7	6.2	1.13 (0.54 to 2.36)
Pulmonary embolism with thrombocytopenia 42 days pre to 14 days post	Vaccinated with BNT162b2 second dose	1,301,209	86,338	5	4.4	1.13 (0.47 to 2.70)
Pulmonary embolism with thrombocytopenia 42 days pre to 14 days post	SARS-CoV-2 PCR positive test	401,571	95,737	12	1.4	8.50 (4.83 to 14.96)
Splanchnic Vein Thrombosis	Vaccinated with ChAdOx1 first dose	3,764,449	277,862	17	14.9	1.14 (0.71 to 1.84)
Splanchnic Vein Thrombosis	Vaccinated with ChAdOx1 second dose	1,090,856	57,967	5	3.9	1.28 (0.53 to 3.07)
Splanchnic Vein Thrombosis	SARS-CoV-2 PCR positive test	401,564	95,736	8	3.1	2.55 (1.27 to 5.09)
Stroke	Vaccinated with ChAdOx1 first dose	3,755,737	277,180	635	759.5	0.84 (0.77 to 0.90)
Stroke	Vaccinated with ChAdOx1 second dose	1,086,113	57,695	216	297.9	0.73 (0.63 to 0.83)
Stroke	Vaccinated with BNT162b2 first dose	1,824,739	138,920	537	637.7	0.84 (0.77 to 0.92)
Stroke	Vaccinated with BNT162b2 second dose	1,296,430	86,003	312	477.7	0.65 (0.58 to 0.73)
Stroke	SARS-CoV-2 PCR positive test	401,062	95,608	121	118.8	1.02 (0.85 to 1.22)
Stroke with thrombocytopenia 10 days pre to 10 days post	Vaccinated with ChAdOx1 first dose	3,764,565	277,870	7	7.1	0.99 (0.47 to 2.08)
Stroke with thrombocytopenia 10 days pre to 10 days post	Vaccinated with BNT162b2 first dose	1,831,463	139,451	5	6.5	0.77 (0.32 to 1.84)
Stroke with thrombocytopenia 42 days pre to 14 days post	Vaccinated with ChAdOx1 first dose	3,764,529	277,868	7	12.5	0.56 (0.27 to 1.17)

Stroke with thrombocytopenia 42 days pre to 14 days post	Vaccinated with ChAdOx1 second dose	1,090,859	57,966	7	5.7	1.22 (0.58 to 2.56)
Stroke with thrombocytopenia 42 days pre to 14 days post	Vaccinated with BNT162b2 first dose	1,831,415	139,448	10	12.1	0.83 (0.44 to 1.54)
Thrombocytopenia	Vaccinated with ChAdOx1 first dose	3,728,941	275,077	2,615	1,966.10	1.33 (1.28 to 1.38)
Thrombocytopenia	Vaccinated with ChAdOx1 second dose	1,070,404	56,790	886	636.3	1.39 (1.30 to 1.49)
Thrombocytopenia	Vaccinated with BNT162b2 first dose	1,801,502	137,106	1,653	1,378.70	1.20 (1.14 to 1.26)
Thrombocytopenia	Vaccinated with BNT162b2 second dose	1,277,216	84,660	1,229	979.4	1.25 (1.19 to 1.33)
Thrombocytopenia	SARS-CoV-2 PCR positive test	399,239	95,143	536	431.7	1.24 (1.14 to 1.35)
Thrombocytopenic purpura	Vaccinated with ChAdOx1 first dose	3,764,491	277,864	31	15.6	1.99 (1.40 to 2.82)
Thrombocytopenic purpura	Vaccinated with BNT162b2 first dose	1,831,387	139,445	9	10	0.90 (0.47 to 1.73)
Thrombocytopenic purpura	SARS-CoV-2 PCR positive test	401,561	95,735	7	4.1	1.70 (0.81 to 3.56)
Venous thromboembolism - broad	Vaccinated with ChAdOx1 first dose	3,750,228	276,749	963	878.6	1.10 (1.03 to 1.17)
Venous thromboembolism - broad	Vaccinated with ChAdOx1 second dose	1,084,594	57,620	229	278.8	0.82 (0.72 to 0.93)
Venous thromboembolism - broad	Vaccinated with BNT162b2 first dose	1,822,257	138,725	642	593.6	1.08 (1.00 to 1.17)
Venous thromboembolism - broad	Vaccinated with BNT162b2 second dose	1,294,840	85,906	361	416.4	0.87 (0.78 to 0.96)
Venous thromboembolism - broad	SARS-CoV-2 PCR positive test	400,648	95,335	1,121	178.2	6.29 (5.93 to 6.67)

Venous thromboembolism - narrow	Vaccinated with ChAdOx1 first dose	3,751,401	276,841	866	819.9	1.06 (0.99 to 1.13)
Venous thromboembolism - narrow	Vaccinated with ChAdOx1 second dose	1,085,037	57,645	211	263.4	0.80 (0.70 to 0.92)
Venous thromboembolism - narrow	Vaccinated with BNT162b2 first dose	1,822,927	138,779	595	560.3	1.06 (0.98 to 1.15)
Venous thromboembolism - narrow	Vaccinated with BNT162b2 second dose	1,295,309	85,938	324	394.7	0.82 (0.74 to 0.92)
Venous thromboembolism - narrow	SARS-CoV-2 PCR positive test	400,723	95,357	1,090	164.3	6.63 (6.25 to 7.04)
Venous thromboembolism broad with thrombocytopenia 10 days pre to 10 days post	Vaccinated with ChAdOx1 first dose	3,764,478	277,863	21	12.7	1.66 (1.08 to 2.54)
Venous thromboembolism broad with thrombocytopenia 10 days pre to 10 days post	Vaccinated with BNT162b2 first dose	1,831,396	139,446	6	8.9	0.67 (0.30 to 1.49)
Venous thromboembolism broad with thrombocytopenia 10 days pre to 10 days post	Vaccinated with BNT162b2 second dose	1,301,175	86,336	10	6.3	1.58 (0.85 to 2.93)
Venous thromboembolism broad with thrombocytopenia 10 days pre to 10 days post	SARS-CoV-2 PCR positive test	401,566	95,736	11	2.1	5.14 (2.85 to 9.28)
Venous thromboembolism broad with thrombocytopenia 42 days pre to 14 days post	Vaccinated with ChAdOx1 first dose	3,764,398	277,857	29	20.4	1.42 (0.99 to 2.05)
Venous thromboembolism broad with thrombocytopenia 42 days pre to 14 days post	Vaccinated with ChAdOx1 second dose	1,090,793	57,963	6	7.2	0.83 (0.37 to 1.84)
Venous thromboembolism broad with thrombocytopenia 42 days pre to 14 days post	Vaccinated with BNT162b2 first dose	1,831,329	139,441	12	14.7	0.81 (0.46 to 1.43)
Venous thromboembolism broad with thrombocytopenia 42 days pre to 14 days post	Vaccinated with BNT162b2 second dose	1,301,119	86,332	16	10.5	1.52 (0.93 to 2.48)
Venous thromboembolism broad with thrombocytopenia 42 days pre to 14 days post	SARS-CoV-2 PCR positive test	401,562	95,735	14	3.4	4.08 (2.41 to 6.88)
Venous thromboembolism narrow with thrombocytopenia 10 days pre to 10 days post	Vaccinated with ChAdOx1 first dose	3,764,482	277,864	16	12.2	1.31 (0.80 to 2.14)

Venous thromboembolism narrow with thrombocytopenia 10 days pre to 10 days post	Vaccinated with BNT162b2 first dose	1,831,401	139,447	6	8.5	0.71 (0.32 to 1.57)
Venous thromboembolism narrow with thrombocytopenia 10 days pre to 10 days post	Vaccinated with BNT162b2 second dose	1,301,179	86,336	10	6	1.67 (0.90 to 3.10)
Venous thromboembolism narrow with thrombocytopenia 10 days pre to 10 days post	SARS-CoV-2 PCR positive test	401,566	95,736	11	2.1	5.34 (2.96 to 9.65)
Venous thromboembolism narrow with thrombocytopenia 42 days pre to 14 days post	Vaccinated with ChAdOx1 first dose	3,764,403	277,858	24	19.6	1.23 (0.82 to 1.83)
Venous thromboembolism narrow with thrombocytopenia 42 days pre to 14 days post	Vaccinated with ChAdOx1 second dose	1,090,796	57,963	5	7	0.72 (0.30 to 1.73)
Venous thromboembolism narrow with thrombocytopenia 42 days pre to 14 days post	Vaccinated with BNT162b2 first dose	1,831,336	139,441	12	14.1	0.85 (0.48 to 1.50)
Venous thromboembolism narrow with thrombocytopenia 42 days pre to 14 days post	Vaccinated with BNT162b2 second dose	1,301,125	86,332	15	10.1	1.49 (0.90 to 2.46)
Venous thromboembolism narrow with thrombocytopenia 42 days pre to 14 days post	SARS-CoV-2 PCR positive test	401,562	95,735	14	3.3	4.27 (2.53 to 7.21)

Supplementary Table 5. Patient profiles: pulmonary embolism

The characteristics of persons with pulmonary embolism used for the primary analyses. *Conditions of interest: autoimmune disease, antiphospholipid syndrome, thrombophilia, asthma, atrial fibrillation, malignant neoplastic disease, diabetes mellitus, obesity, or renal impairment. †Medications of interest included non-steroidal anti-inflammatory drugs, Cox2 inhibitors, systemic corticosteroids, hormonal contraceptives, tamoxifen, and sex hormones and modulators of the genital system

	**ChAdOx1 first dose**	**ChAdOx1 second dose**	**BNT162b2 first dose**	**BNT162b2 second dose**	**SARS-CoV-2 PCR positive test**	**General population**
N	510	106	352	169	919	28,454
Age	67 [57 to 77]	75 [71 to 81]	73 [62 to 81]	81 [74 to 85]	58 [49 to 67]	68 [55 to 78]
Age: 20 to 29	14 (2.7%)	0 (0.0%)	6 (1.7%)	<5	25 (2.7%)	868 (3.1%)
Age: 30 to 39	13 (2.5%)	0 (0.0%)	9 (2.6%)	<5	83 (9.0%)	1,566 (5.5%)
Age: 40 to 49	36 (7.1%)	<5	21 (6.0%)	6 (3.6%)	134 (14.6%)	2,644 (9.3%)
Age: 50 to 59	91 (17.8%)	7 (6.6%)	34 (9.7%)	10 (5.9%)	266 (28.9%)	4,358 (15.3%)
Age: 60 to 69	126 (24.7%)	11 (10.4%)	69 (19.6%)	10 (5.9%)	232 (25.2%)	5,996 (21.1%)
Age: 70 to 79	142 (27.8%)	53 (50.0%)	102 (29.0%)	36 (21.3%)	131 (14.3%)	7,294 (25.6%)
Age: 80 or older	88 (17.3%)	33 (31.1%)	111 (31.5%)	99 (58.6%)	48 (5.2%)	5,728 (20.1%)
Sex: Male	252 (49.4%)	52 (49.1%)	164 (46.6%)	75 (44.4%)	553 (60.2%)	13,538 (47.6%)
Years of prior observation time	14.0 [5.0 to 30.6]	18.9 [5.3 to 34.0]	20.6 [8.5 to 34.1]	24.2 [7.5 to 37.7]	14.2 [6.2 to 26.6]	18.3 [7.0 to 29.2]
Comorbidities						
Autoimmune disease	19 (3.7%)	7 (6.6%)	20 (5.7%)	13 (7.7%)	34 (3.7%)	1,286 (4.5%)
Antiphospholipid syndrome	0 (0.0%)	0 (0.0%)	0 (0.0%)	0 (0.0%)	<5	86 (0.3%)
Thrombophilia	<5	<5	<5	0 (0.0%)	<5	223 (0.8%)
Asthma	82 (16.1%)	16 (15.1%)	55 (15.6%)	35 (20.7%)	162 (17.6%)	4,630 (16.3%)
Atrial fibrillation	27 (5.3%)	6 (5.7%)	14 (4.0%)	16 (9.5%)	10 (1.1%)	1,235 (4.3%)
Malignant neoplastic disease	127 (24.9%)	44 (41.5%)	111 (31.5%)	76 (45.0%)	88 (9.6%)	5,774 (20.3%)
Diabetes mellitus	81 (15.9%)	11 (10.4%)	52 (14.8%)	27 (16.0%)	165 (18.0%)	3,967 (13.9%)
Obesity	44 (8.6%)	8 (7.5%)	23 (6.5%)	13 (7.7%)	100 (10.9%)	2,415 (8.5%)

Heart disease	104 (20.4%)	26 (24.5%)	76 (21.6%)	59 (34.9%)	112 (12.2%)	5,629 (19.8%)
Hypertensive disorder	183 (35.9%)	54 (50.9%)	157 (44.6%)	82 (48.5%)	272 (29.6%)	11,304 (39.7%)
Renal impairment	81 (15.9%)	36 (34.0%)	81 (23.0%)	49 (29.0%)	82 (8.9%)	4,569 (16.1%)
COPD	42 (8.2%)	13 (12.3%)	40 (11.4%)	21 (12.4%)	37 (4.0%)	2,674 (9.4%)
Dementia	25 (4.9%)	10 (9.4%)	12 (3.4%)	11 (6.5%)	21 (2.3%)	708 (2.5%)
Medication use (183 days prior to four days prior)						
Non-steroidal anti-inflammatory drugs	119 (23.3%)	27 (25.5%)	86 (24.4%)	48 (28.4%)	158 (17.2%)	7,065 (24.8%)
Cox2 inhibitors	<5	0 (0.0%)	<5	0 (0.0%)	0 (0.0%)	30 (0.1%)
Systemic corticosteroids	58 (11.4%)	13 (12.3%)	35 (9.9%)	26 (15.4%)	63 (6.9%)	3,294 (11.6%)
Antithrombotic and anticoagulant therapies	31 (6.1%)	6 (5.7%)	28 (8.0%)	13 (7.7%)	30 (3.3%)	1,931 (6.8%)
Lipid modifying agents	29 (5.7%)	7 (6.6%)	22 (6.2%)	12 (7.1%)	51 (5.5%)	1,892 (6.6%)
Antineoplastic and immunomodulating agents	12 (2.4%)	0 (0.0%)	8 (2.3%)	<5	11 (1.2%)	512 (1.8%)
Hormonal contraceptives for systemic use	7 (1.4%)	0 (0.0%)	5 (1.4%)	0 (0.0%)	7 (0.8%)	323 (1.1%)
Tamoxifen	<5	0 (0.0%)	<5	0 (0.0%)	<5	29 (0.1%)
Sex hormones and modulators of the genital system	14 (2.7%)	0 (0.0%)	8 (2.3%)	<5	15 (1.6%)	570 (2.0%)
One or more condition of interest*	249 (48.8%)	76 (71.7%)	206 (58.5%)	118 (69.8%)	337 (36.7%)	13,564 (47.7%)
One or more medication of interest†	149 (29.2%)	32 (30.2%)	107 (30.4%)	57 (33.7%)	197 (21.4%)	8,475 (29.8%)
One or more condition/ medication of interest*†	307 (60.2%)	83 (78.3%)	241 (68.5%)	131 (77.5%)	441 (48.0%)	17,284 (60.7%)

Supplementary Table 6. Patient profiles: cerebral venous sinus thrombosis

The characteristics of persons with pulmonary embolism used for the primary analyses. *Conditions of interest: autoimmune disease, antiphospholipid syndrome, thrombophilia, asthma, atrial fibrillation, malignant neoplastic disease, diabetes mellitus, obesity, or renal impairment. †Medications of interest included non-steroidal anti-inflammatory drugs, Cox2 inhibitors, systemic corticosteroids, hormonal contraceptives, tamoxifen, and sex hormones and modulators of the genital system

	**ChAdOx1 first dose**	**SARS-CoV-2 PCR positive test**	**General population**
N	21	5	382
Age	47 [30 to 58]	52 [40 to 67]	48 [35 to 64]
Age: 20 to 29	5 (23.8%)	0 (0.0%)	56 (14.7%)
Age: 30 to 39	<5	<5	68 (17.8%)
Age: 40 to 49	<5	<5	81 (21.2%)
Age: 50 to 59	5 (23.8%)	<5	60 (15.7%)
Age: 60 to 69	<5	<5	56 (14.7%)
Age: 70 to 79	<5	0 (0.0%)	35 (9.2%)
Age: 80 or older	<5	<5	26 (6.8%)
Sex: Male	11 (52.4%)	<5	145 (38.0%)
Years of prior observation time	9.8 [2.1 to 28.5]	30.1 [24.1 to 33.8]	12.4 [4.2 to 24.1]
Comorbidities	NA	NA	NA
Autoimmune disease	0 (0.0%)	0 (0.0%)	16 (4.2%)
Antiphospholipid syndrome	0 (0.0%)	0 (0.0%)	<5
Thrombophilia	0 (0.0%)	0 (0.0%)	7 (1.8%)
Asthma	5 (23.8%)	0 (0.0%)	72 (18.8%)
Atrial fibrillation	0 (0.0%)	0 (0.0%)	7 (1.8%)
Malignant neoplastic disease	0 (0.0%)	<5	37 (9.7%)
Diabetes mellitus	<5	<5	30 (7.9%)
Obesity	<5	<5	25 (6.5%)
Heart disease	<5	<5	32 (8.4%)
Hypertensive disorder	5 (23.8%)	<5	84 (22.0%)

Renal impairment	0 (0.0%)	0 (0.0%)	22 (5.8%)
COPD	0 (0.0%)	<5	8 (2.1%)
Dementia	<5	0 (0.0%)	<5
Medication use (183 days prior to four days prior)	NA	NA	NA
Non-steroidal anti-inflammatory drugs	<5	<5	65 (17.0%)
Cox2 inhibitors	0 (0.0%)	0 (0.0%)	<5
Systemic corticosteroids	0 (0.0%)	<5	27 (7.1%)
Antithrombotic and anticoagulant therapies	<5	<5	23 (6.0%)
Lipid modifying agents	<5	0 (0.0%)	14 (3.7%)
Antineoplastic and immunomodulating agents	0 (0.0%)	0 (0.0%)	13 (3.4%)
Hormonal contraceptives for systemic use	0 (0.0%)	0 (0.0%)	13 (3.4%)
Tamoxifen	0 (0.0%)	0 (0.0%)	0 (0.0%)
Sex hormones and modulators of the genital system	0 (0.0%)	0 (0.0%)	17 (4.5%)
One or more condition of interest	<5	<5	102 (26.7%)
One or more medication of interest	<5	<5	84 (22.0%)
One or more condition/ medication of interest	<5	<5	156 (40.8%)

Supplementary Table 7. Patient profiles: thrombocytopenia

The characteristics of persons with thrombocytopenia used for the primary analyses. *Conditions of interest: autoimmune disease, antiphospholipid syndrome, thrombophilia, asthma, atrial fibrillation, malignant neoplastic disease, diabetes mellitus, obesity, or renal impairment. †Medications of interest included non-steroidal anti-inflammatory drugs, Cox2 inhibitors, systemic corticosteroids, hormonal contraceptives, tamoxifen, and sex hormones and modulators of the genital system

	**ChAdOx1 first dose**	**ChAdOx1 second dose**	**BNT162b2 first dose**	**BNT162b2 second dose**	**SARS-CoV-2 PCR positive test**	**General population**
N	2,900	959	1,769	1,296	598	148,393
Age	66 [55 to 76]	76 [70 to 81]	77 [64 to 84]	81 [74 to 86]	55 [40 to 66]	65 [49 to 77]
Age: 20 to 29	77 (2.7%)	8 (0.8%)	20 (1.1%)	11 (0.8%)	55 (9.2%)	8,676 (5.8%)
Age: 30 to 39	127 (4.4%)	16 (1.7%)	38 (2.1%)	20 (1.5%)	87 (14.5%)	12,728 (8.6%)
Age: 40 to 49	268 (9.2%)	26 (2.7%)	93 (5.3%)	40 (3.1%)	84 (14.0%)	15,896 (10.7%)
Age: 50 to 59	547 (18.9%)	49 (5.1%)	186 (10.5%)	47 (3.6%)	133 (22.2%)	22,224 (15.0%)
Age: 60 to 69	635 (21.9%)	128 (13.3%)	246 (13.9%)	104 (8.0%)	113 (18.9%)	26,642 (18.0%)
Age: 70 to 79	789 (27.2%)	440 (45.9%)	448 (25.3%)	321 (24.8%)	72 (12.0%)	32,357 (21.8%)
Age: 80 or older	457 (15.8%)	292 (30.4%)	738 (41.7%)	753 (58.1%)	54 (9.0%)	29,870 (20.1%)
Sex: Male	1,808 (62.3%)	615 (64.1%)	1,164 (65.8%)	836 (64.5%)	353 (59.0%)	89,252 (60.1%)
Years of prior observation time	14.9 [4.6 to 28.3]	19.5 [5.9 to 32.5]	21.1 [6.8 to 32.9]	22.7 [9.0 to 34.0]	12.0 [4.0 to 23.3]	16.2 [5.7 to 27.6]
Comorbidities						
Autoimmune disease	191 (6.6%)	62 (6.5%)	147 (8.3%)	77 (5.9%)	36 (6.0%)	7,290 (4.9%)
Antiphospholipid syndrome	5 (0.2%)	<5	5 (0.3%)	<5	<5	194 (0.1%)
Thrombophilia	10 (0.3%)	<5	7 (0.4%)	<5	<5	344 (0.2%)

Asthma	365 (12.6%)	119 (12.4%)	204 (11.5%)	156 (12.0%)	75 (12.5%)	17,613 (11.9%)
Atrial fibrillation	378 (13.0%)	203 (21.2%)	441 (24.9%)	339 (26.2%)	36 (6.0%)	16,320 (11.0%)
Malignant neoplastic disease	530 (18.3%)	264 (27.5%)	485 (27.4%)	409 (31.6%)	64 (10.7%)	24,193 (16.3%)
Diabetes mellitus	709 (24.4%)	284 (29.6%)	480 (27.1%)	335 (25.8%)	127 (21.2%)	25,804 (17.4%)
Obesity	245 (8.4%)	80 (8.3%)	108 (6.1%)	74 (5.7%)	48 (8.0%)	8,576 (5.8%)
Heart disease	932 (32.1%)	428 (44.6%)	838 (47.4%)	720 (55.6%)	122 (20.4%)	40,270 (27.1%)
Hypertensive disorder	1,280 (44.1%)	531 (55.4%)	989 (55.9%)	806 (62.2%)	186 (31.1%)	57,802 (39.0%)
Renal impairment	545 (18.8%)	270 (28.2%)	543 (30.7%)	457 (35.3%)	75 (12.5%)	25,118 (16.9%)
COPD	199 (6.9%)	74 (7.7%)	149 (8.4%)	140 (10.8%)	13 (2.2%)	8,220 (5.5%)
Dementia	122 (4.2%)	53 (5.5%)	65 (3.7%)	65 (5.0%)	23 (3.8%)	3,398 (2.3%)
Medication use (183 days prior to four days prior)						
Non-steroidal anti-inflammatory drugs	498 (17.2%)	191 (19.9%)	361 (20.4%)	245 (18.9%)	90 (15.1%)	30,345 (20.4%)
Cox2 inhibitors	<5	0 (0.0%)	<5	<5	0 (0.0%)	149 (0.1%)
Systemic corticosteroids	224 (7.7%)	89 (9.3%)	138 (7.8%)	105 (8.1%)	44 (7.4%)	12,972 (8.7%)
Antithrombotic and anticoagulant therapies	152 (5.2%)	75 (7.8%)	168 (9.5%)	148 (11.4%)	22 (3.7%)	9,562 (6.4%)
Lipid modifying agents	209 (7.2%)	89 (9.3%)	187 (10.6%)	157 (12.1%)	39 (6.5%)	10,163 (6.8%)
Antineoplastic and immunomodulating agents	43 (1.5%)	13 (1.4%)	24 (1.4%)	19 (1.5%)	8 (1.3%)	2,459 (1.7%)
Hormonal contraceptives for systemic use	27 (0.9%)	<5	8 (0.5%)	<5	5 (0.8%)	1,912 (1.3%)

Tamoxifen	<5	<5	<5	0 (0.0%)	0 (0.0%)	81 (0.1%)
Sex hormones and modulators of the genital system	54 (1.9%)	8 (0.8%)	23 (1.3%)	9 (0.7%)	12 (2.0%)	2,875 (1.9%)
One or more condition of interest	1,633 (56.3%)	697 (72.7%)	1,295 (73.2%)	977 (75.4%)	254 (42.5%)	70,469 (47.5%)
One or more medication of interest	628 (21.7%)	228 (23.8%)	430 (24.3%)	293 (22.6%)	117 (19.6%)	36,867 (24.8%)
One or more condition/ medication of interest	1,861 (64.2%)	750 (78.2%)	1,409 (79.6%)	1,034 (79.8%)	301 (50.3%)	86,443 (58.3%)
